# Electrically programmable organic in-display neuromorphic computing

**DOI:** 10.1093/nsr/nwaf224

**Published:** 2025-06-04

**Authors:** Shilei Dai, Dingchen Wang, Xu Liu, Binbin Cui, Xinyu Tian, Dingyao Liu, Huawei Hu, Pu Guo, Tongrui Sun, Junyao Zhang, Jia Huang, Shiming Zhang, Zhongrui Wang

**Affiliations:** Department of Electrical and Electronic Engineering, The University of Hong Kong, Hong Kong 999077, China; Department of Electrical and Electronic Engineering, The University of Hong Kong, Hong Kong 999077, China; School of Microelectronics, Southern University of Science and Technology, Shenzhen 518055, China; School of Materials Science and Engineering, Tongji University, Shanghai 201804, China; Department of Electrical and Electronic Engineering, The University of Hong Kong, Hong Kong 999077, China; Department of Electrical and Electronic Engineering, The University of Hong Kong, Hong Kong 999077, China; Department of Electrical and Electronic Engineering, The University of Hong Kong, Hong Kong 999077, China; State Key Laboratory for Modification of Chemical Fibers and Polymer Materials & College of Materials Science and Engineering, Donghua University, Shanghai 201620, China; School of Materials Science and Engineering, Tongji University, Shanghai 201804, China; School of Materials Science and Engineering, Tongji University, Shanghai 201804, China; School of Materials Science and Engineering, Tongji University, Shanghai 201804, China; School of Materials Science and Engineering, Tongji University, Shanghai 201804, China; Department of Electrical and Electronic Engineering, The University of Hong Kong, Hong Kong 999077, China; Department of Electrical and Electronic Engineering, The University of Hong Kong, Hong Kong 999077, China; School of Microelectronics, Southern University of Science and Technology, Shenzhen 518055, China

**Keywords:** organic electronics, neuromorphic devices, in-display computing, reconfigurable device

## Abstract

The scale-up of AI models and data makes smart edge devices (e.g. smartphones and AR/VR glasses) face increasing challenges in energy consumption, latency and hardware costs. These challenges are due to their physically separated memory, processing and display units. Collocating the functions of these units into a single device for in-display neuromorphic computing (IDNC) shows great promise in overcoming these challenges. Herein, we report an all-in-one electrically programmable IDNC (EP-IDNC) device that leverages the electrochromism of organic semiconductors. The electrochromism empowers the EP-IDNC device with synaptic and neural behaviors (e.g. multi-terminal operability, multi-level weight update, spatiotemporal information encoding), as well as the capability to visually display computing results through color changes. The IDNC functions are validated on tasks such as noise reduction and motion object perception using a prototype 3 × 3 EP-IDNC device array. We ultimately showcase the integration of neuromorphic and display functions for car steering reminders.

## Teaser

This work reports an electrically programmable in-display neuromorphic computing device that leverages the electrochromism of organic semiconductors to simultaneously process information like the human brain while visually displaying computing results.

## INTRODUCTION

**Figure 1.**
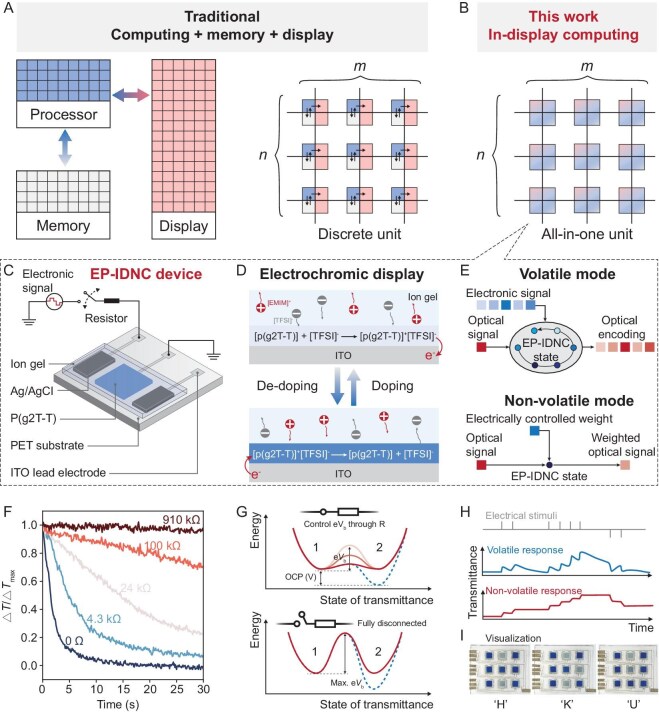
Operation principle and device structure of the EP-IDNC device. (A and B) Schematic diagrams comparing (A) traditional display and computing technology with (B) in-display neuromorphic computing. (C) Schematic diagram of the reconfigurable and multi-terminal EP-IDNC device. (D) Working mechanism of the EP-IDNC device as a display unit. The color of the p(g2T-T) layer can be regulated by applying a voltage to the Ag/AgCl electrode to drive the electrochemical reaction. (E) The schematic illustrates the utilization of volatile and non-volatile modes in the EP-IDNC device to achieve a variety of computing functions, with electrically controlled color (i.e. transmittance) acting as a synaptic weight. (F) EP-IDNC device state retention control is achieved by varying the series resistor value. (G) Schematic explaining the state retention control with resistors of different values. (H) Transmittance responses of the EP-IDNC device working in volatile mode (top panel) and non-volatile mode (bottom panel). (I) The prototype two-terminal EP-IDNC device array displaying the initials of ‘Hong Kong University’, i.e. HKU.

Smart edge systems, such as augmented reality (AR)/virtual reality (VR) glasses, smartphones and watches, are experiencing unprecedented changes amid the scale-up of data and machine learning models, where displays are crucial in human–device interaction [1]. However, these systems are primarily built on the CMOS digital platforms, featuring von Neumann architecture with physically separated memory and processing, as well as display units (Fig. 1A). The frequent data shuttling among these units and unavoidable sequential analog–digital conversions lead to high energy consumption and notable delays [2–4]. Additionally, the heterogeneous integration of these functional units requires complex manufacturing processes.

In-display neuromorphic computing (IDNC) offers a promising solution to address these challenges by collocating memory, processing and display functions on a single all-in-one device (Fig. 1B). Neuromorphic computing results in a significant reduction in energy and latency by minimizing data transmission pathways and decreasing power consumption associated with data movement [2,3]. Additionally, merging computing and display avoids frequent analog–digital signal conversion, leading to swift real-time display and enhanced human interaction [5]. Furthermore, it will substantially reduce the cost of smart edge devices and simplify integration complexity through homogeneous integration. The implementation of IDNC requires an all-in-one device with several essential features, including: (i) synaptic and neuronal functions—such as multi-terminal operation, short-/long-term (i.e. volatile/non-volatile) memory (STM/LTM)—to enable brain-inspired information encoding [6,7], integration [8] and in-memory processing [9,10]; and (ii) visualization capability to showcase computing results in real time.

Electrical neuromorphic devices, such as memristors [6,11–18], phase-change memories [19–21], ferroelectric memories [22–24], neuromorphic transistors [25–36] and electrochemical random-access memories (ECRAMs) [9,37–39], have replicated vast amounts of synaptic and neuronal functions. However, the absence of display capability in these devices hinders their application in IDNC. On the other hand, photonic neuromorphic devices [40–47] show great potential in signal processing by offering low latency, high bandwidth and exceptional energy efficiency. Nevertheless, optical signals used in these devices typically fall outside the human visible range [44,48,49]. Moreover, due to the limitations of the underlying mechanism, pure photonic neuromorphic devices struggle to accurately emulate crucial synaptic and neural behaviors (e.g. multi-terminal operability and volatile memory) [48,49] (Table S1). Neuromorphic light-emitting devices have recently emerged as promising platforms for neuromorphic displays, synergistically integrating synaptic computation and visible-light emission [4,50,51]. While these pioneering studies demonstrate groundbreaking potential for IDNC, current implementations remain constrained by the absence of multi-terminal operability and reconfigurability.

Therefore, the integration of memory, computing and display capabilities, as well as multi-terminal operability and reconfigurability into a futuristic all-in-one device for IDNC is yet to be demonstrated. The major gap lies in the absence of a suitable device platform with a unified physics for implementing all these functions.

Herein, we exploit the electrochromism in organic semiconductors (OSCs) to devise the all-in-one electrically programmable IDNC (EP-IDNC) devices (Fig. 1C–I). The electrochromism, extensively explored for next-generation displays [1], enables simultaneous neuromorphic computing and display. Specifically, the electrochromic color functions as both the display tool for visualizing computing results (Fig. 1D) and the synaptic weight for signal processing (Fig. 1E). For neuromorphic behavior, the programming of the EP-IDNC device, akin to battery charging/discharging [52], allows for easy configuration of STM and LTM by precisely controlling the discharging rate (Fig. 1F and G). Multi-terminal EP-IDNCs are also realized through ionic–electrochromic coupling effects, which opens the possibility of integrating various external signals into a single device, akin to biological dendrites (Fig. 1C). We initially assessed the STM and multi-terminal operability of the EP-IDNC device for spatiotemporal information encoding/processing, followed by testing the LTM behavior of the device for in-memory computing. Subsequently, the IDNC function was validated on tasks such as noise reduction and motion object perception using a prototype 3 × 3 EP-IDNC device array. During the final demonstration of the IDNC application for a car steering reminder, we utilized the multi-terminal feature of the EP-IDNC device to fuse analog reservoir states and synaptic weight for information encoding and processing (e.g. classification) within the same EP-IDNC device array. Furthermore, we utilized the EP-IDNC device itself within the array as the display pixel to showcase computing results in real time.

## RESULTS

### Device design and working mechanism characterization

As shown in Fig. 1C, the multi-terminal EP-IDNC device is composed of five functional components: (i) ion gel layer; (ii) electrochromic layer; (iii) silver/silver chloride (Ag/AgCl) electrode; (iv) indium tin oxide (ITO) lead electrode; and (v) ultra-transparent polyethylene glycol terephthalate (PET) substrate. The OSC, namely poly(2-(3,3′-bis(2-(2-(2-methoxyethoxy)ethoxy)ethoxy)-[2,2′-bithiophen]-5)yl thiophene) (p(g2T-T)) was chosen as the electrochromic layer because of its excellent electrochemical properties, low-cost solution processability and very reliable performance (Fig. S1) [53]. The Ag/AgCl paste is used as the counter electrode because it is a non-polarizable electrode [54]. The number of input terminals on the EP-IDNC devices is determined by the quantity of identical Ag/AgCl counter electrodes used. For example, Fig. 1C displays a triple-terminal EP-IDNC device with two identical input terminals and one ground terminal. Photo-patternable ion gels [55] are employed to realize solid-state devices with well-patterned electrolyte layers and high switching contrast (Figs S2 and S3). In addition, the high ionic conductivity (∼5 mS/cm) and high capacitance (>1 μF/cm^2^ at 1 kHz) of the ion gel can lead to the low-voltage operation of the EP-IDNC device (<1 V) (Fig. S4). PET-ITO is used as the lead electrode and substrate simultaneously because of its high transparency (>85% at 600 nm) (Fig. S5).

The electrochromic property of p(g2T-T) is characterized by using ultraviolet-visible spectroscopy (UV-vis) (Fig. S6). Notably, as the control voltage changes from 1 to −1 V (i.e. applied to Ag/AgCl), the absorption in the 600–650 nm band gradually decreases. Meanwhile, a new spectral band appears near 920 nm. The new spectral band first increases and then decreases with the change in the control voltage. The UV-vis result indicates that p(g2T-T) exhibits different transmittance responses at different wavelengths concerning control voltage. Electrostatically neutral ion pairs (i.e. [EMIM]^+^[TFSI]^−^) within the ion gel layer can permeate the p(g2T-T) film in both passive diffusion and active field-driven migration under applied voltages [56]. These permeated ions solvate the polar ethylene glycol (EG) side chains via hydrogen bonding and dipole-ion coordination with oxygen atoms in the EG units [57]. This process subsequently facilitates electrochemical doping and de-doping by altering the ionic composition within the film. As illustrated in Fig. S7, applying a positive voltage to the counter electrode extracts anions from the p(g2T-T) semiconductor, while cations are introduced into the semiconductor, leading to de-doping. Conversely, applying a negative voltage to the counter electrode introduces anions into the semiconductor to compensate for holes on the conjugated chain, while extracting cations from the semiconductor film, resulting in doping. The doping/de-doping process is highly reversible, as evidenced by the cyclic voltammogram (CV) curve of p(g2T-T) (Fig. S8). Upon doping, with different positive charge-carrying states on the main chain of p(g2T-T) polymer, polarons and/or bipolarons are formed (Fig. S9A–C) [58,59]. The UV-vis spectral changes of p(g2T-T) upon doping can be explained as follows: when a polaron is formed, two localized electronic states, +*ω*_0_ and −*ω*_0_, are generated in the band gap of undoped (neutral) p(g2T-T), as shown in Fig. S9D. The 600–650 nm absorption band is related to the optical band gap of p(g2T-T), while the absorption band at 920 nm can be assigned to the polaron state transition P2 from +*ω*_0_ to −*ω*_0_. When the doping level increases, the polaronic absorption increases accordingly, which bleaches the neutral state absorption (600–650 nm). At large negative voltage, high doping levels can be achieved, resulting in the formation of bipolarons. In this case, two new localized electronic states, +ω_0_′ and −ω_0_′, are generated. The ω_0_′ level of the bipolarons is deeper than the corresponding ω_0_ level of the polarons. The decrease of the absorption in the 920 nm band is due to the polarons being gradually transferred to bipolarons under large doping voltage, which will cause the increase of the absorption band above 1100 nm (Fig. S6).

The p(g2T-T) electrochromic layer can be switched between a transparent (or oxidized) state and an opaque state (or reduced state) at 600–650 nm by simply changing the polarity of the external power source (Fig. 1D). The color (i.e. transmittance) of the device has been regarded as the synaptic weight so that light can be used to multiply with the electrically controlled weight to realize computing or spatiotemporal information encoding in the optical domain (Fig. 1E). Bidirectional weight modulation is possible because the p(g2T-T) film is moderately doped when zero-voltage bias is applied to the Ag/AgCl electrode. Notably, the voltage-controlled doping/de-doping process mirrors the charging/discharging mechanism of a battery [52]. Therefore, precise control of the discharging rate enables the device to exhibit both volatile and non-volatile memory characteristics (see Note S1). To validate our concept, we connect a resistor in series to the EP-IDNC device (Fig. 1C and Fig. S10). After programming, as the resistance value of the series resistor increases, the weight retention time also increases (Fig. 1F). Within the appropriate resistance range, tunable STM can be achieved by adjusting the resistance value to modulate the discharging barrier (e*V*_b_) (Fig. 1G (top panel)). To achieve non-volatile behavior, external circuits can be disconnected after programming through physical switches to achieve the maximum discharge barrier (max. e*V*_b_) (Fig. 1G (bottom panel)), which is very similar to the working mechanism of the emerging ECRAMs [9,37,38,60]. Therefore, both volatile and non-volatile properties can be achieved in a single EP-IDNC device (Fig. 1H). It is also worth noting that our device features multiple identical terminals, allowing each terminal to be configured independently between volatile and non-volatile modes. More importantly, the EP-IDNC device array is also capable of serving as a display (Fig. 1I) and facilitating the implementation of the IDNC.

### Volatile neuromorphic behavior

**Figure 2.**
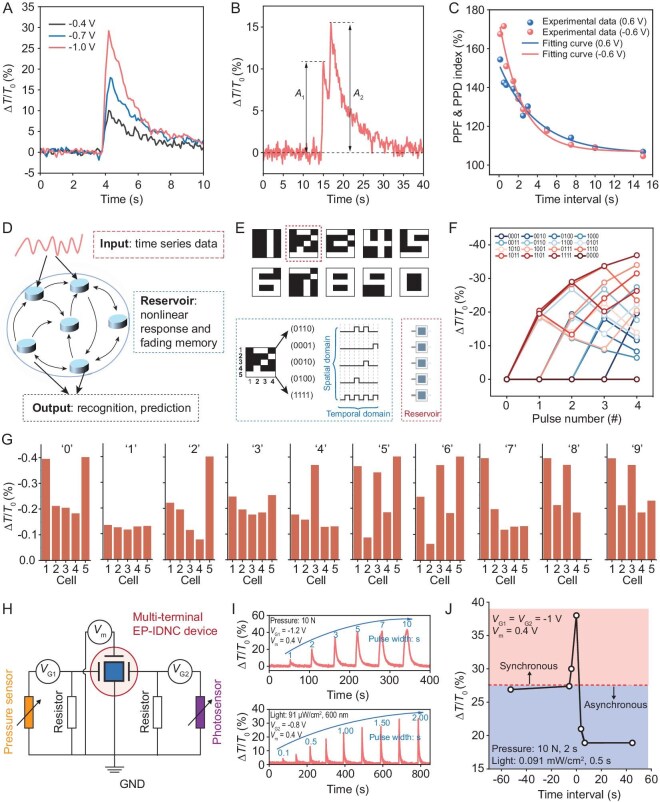
Volatile neuromorphic performance of the EP-IDNC device. (A and B) EPSP of the EP-IDNC device triggered by (A) one voltage spike, and (B) two sequential voltage spikes. (C) The experimental PPF and PPD index as functions of the time interval between the two peaks in (B) and their fitting curves. (D) Schematic illustration of the RC network. (E) Images of 10 digits used in this test, and the corresponding voltage stream generation principle based on these digits. (F) Reservoir states of the EP-IDNC device used to differentiate different temporal inputs. (G) Experimentally measured reservoir states of five EP-IDNC devices subjected to the 10 inputs. (H) Circuit diagram for the implementation of visual-haptic fusion function by using 4-terminal EP-IDNC devices. *V*_G1_ and *V*_G2_ serve as voltage biases used to convert the sensor response into voltage responses applied to the two programming gate terminals of the EP-IDNC device, while *V*_m_ is the voltage bias applied to the modulation terminal of the EP-IDNC device. (I) Responses of the multi-terminal EP-IDNC device in (H) to pressure stimuli (top panel) and light stimuli (bottom panel), respectively. (J) Plot of relative transmittance changes of multi-terminal EP-IDNC device in (H) as a function of the time interval between pressure stimuli and light stimuli. In all, 27.5% is defined as the criterion of synchronization of the two sensor signals.

The relative transmittance change of the EP-IDNC device can be temporarily altered by applying voltage spikes. After programming, the EP-IDNC device exhibits a non-linear fading memory effect, which is similar to the excitatory post-synaptic potential (EPSP)/inhibitory post-synaptic potential (IPSP) behavior in a bio-synapse (Fig. 2A, Fig. S11) [27,61,62]. The response and memory retention time increase with the increase of control voltage, as the variation in doping level increases more with the increase of pulse voltage, resulting in a more pronounced change in transmittance. After programming, due to the potential imbalance between the electrochromic layer and the control gate, the EP-IDNC device will self-discharge to balance the potential, resulting in non-linear memory decay behavior. The EPSP/IPSP of the EP-IDNC device can also be modulated by changing the pulse width (Fig. S12). Other typical STM behaviors like paired-pulse facilitation (PPF) and paired-pulse depression (PPD) are also achieved (Fig. 2B and C; Figs S13 and S14). When the second pulse voltage arrives before the relaxation of doping/de-doping changes induced by the first pulse voltage, the accumulative effect leads to a higher transmittance response, thus forming the PPF/PPD behavior of EP-IDNC devices. Such cumulative effects in EP-IDNC devices can also be naturally used to realize other types of plasticity, such as spike-rate-dependent plasticity (SRDP) (Figs S15 and S16).

To demonstrate the potential of utilizing the volatile memory of the EP-IDNC device for information encoding and processing, we employed the EP-IDNC device to implement reservoir computing (RC). RC is a best-in-class neural network-based AI algorithm for processing time-series data generated in non-linear dynamic systems (Fig. 2D). The hardware implementation of RC systems requires two main characteristics of primitives: (i) exhibiting non-linear response in the presence of temporal input signals; and (ii) displaying fading memory, to map the inputs to a new space, and performing non-linear transformation of the input information. Given it features a non-linear response and fading memory, our EP-IDNC device is well-suited for implementing RC. To demonstrate this concept, we use an RC system consisting of five EP-IDNC devices to recognize digital images, with each image consisting of 20 pixels, either white (1) or black (0) (Fig. 2E). During classification, each of the images has been divided into five rows containing four consecutive pixels. Based on the color of the pixel, a 4-timeframe voltage input stream is generated and fed into the EP-IDNC device in the same row for image feature extraction. In our experiment, if the pixel color is black, the input voltage is 0 V (1 s); otherwise, the input voltage is 0.7 V and the duty cycle is 50% (1 s). Our EP-IDNC device can have 16 distinct RC states for 4-bit sequential voltage inputs, ranging from ‘0000’ to ‘1111’, thanks to its excellent non-linear response and volatile behavior (Fig. 2F). Moreover, all of the five EP-IDNC devices used in digital image recognition demonstrate similar RC behavior, which can be evidenced by the response of each device to the 4-bit inputs (Fig. S17). As the transmittance states of EP-IDNCs are dependent on the pattern of temporal inputs, after the application of the five input voltage streams generated based on each digital image to the RC system (Fig. 2E), a distinguishable output transmittance state for each EP-IDNC device of our RC system can be observed, verifying the ability of the EP-IDNC-based reservoir to separate the 10 digital images (Fig. 2G). It is worth noting that the RC results can also be adjusted by changing the thickness of the electrochromic layer or the parameters of the pulse (Fig. S18). The simulation results show that our EP-IDNC-based RC system can achieve an accuracy of 100% for the 10 digital images in the software-based readout layer, further verifying its suitability for time-series signal processing (Fig. S19).

Next, we demonstrated utilizing the volatile memory and multi-terminal features of EP-IDNC devices for information processing. The multi-terminal EP-IDNC can not only mimic the dendrite structure of neurons but also enable the EP-IDNC to be capable of implementing more complex functions, such as integrating signals from different sensors. As a proof of concept, we fabricated a 4-terminal EP-IDNC device and interfaced it with a pressure sensor and a photodetector to demonstrate the visual-haptic fusion function (Fig. 2H and Fig. S20). The remaining terminal is used as the modulation port. The information flow in each sensing-computing path starts from triggering the specific sensor in response to external stimuli (Fig. 2I; Figs S21 and S22). The resistance of each sensor decreases under stimulation, so the voltage source triggers ion flux in the ion storage layer, resulting in a change in the transmittance. We demonstrate visual-haptic synchronization by triggering two sensors at different time intervals. As shown in Fig. 2J, bimodal sensory stimuli in closer succession can induce stronger relative transmittance changes of EP-IDNC devices, which can be used to determine the degree of synchronization between two sensory signals. Also, the stronger relative transmittance changes caused by bimodal sensory stimuli enable EP-IDNC devices to tolerate a certain amount of time difference between two signals while still considering them to be synchronized. The achieved visual-haptic fusion function is expected to be used in application scenarios such as robotic arm control [8].

### Non-volatile neuromorphic behavior

**Figure 3.**
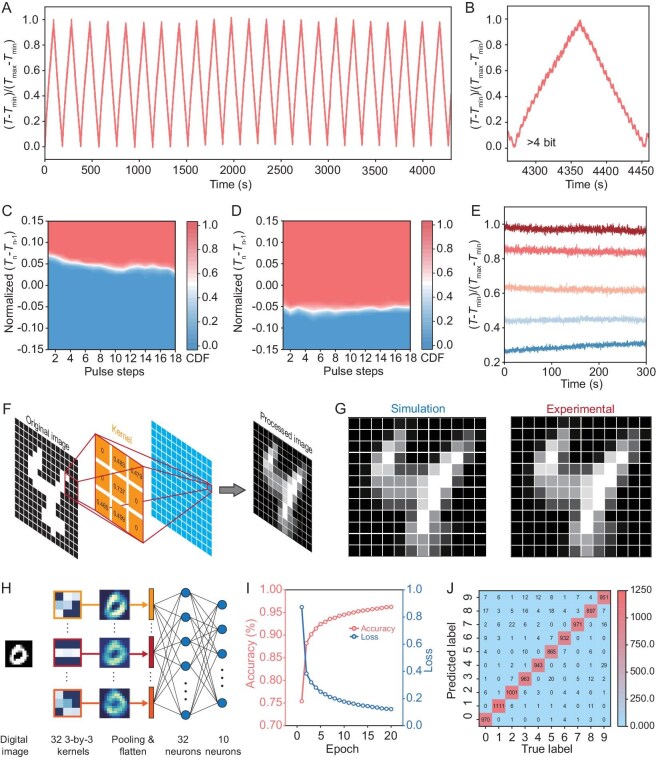
Non-volatile neuromorphic performance of the EP-IDNC device. (A) LTP-LTD curves of the EP-IDNC device, showing excellent cyclic stability and weight updating linearity. (B) More than 4 bits of discrete memory states have been achieved in non-volatile EP-IDNC devices. (C and D) The CDF heatmaps show the extracted per-spike normalized transmittance change at each pulse step in (C) potentiation and (D) depression. (E) State-retention curves for non-volatile EP-IDNC device left at open-circuit for 5 min after programming to a particular state. (F) A schematic showing the non-volatile EP-IDNC device serves as the kernel in photonic CNNs. (G) Edge detection of the digital number ‘4’ using a non-volatile EP-IDNC device to construct the photonic kernel layer. Left: simulation results; right: experimental results. (H) Schematic of the simulated photonic CNN for handwritten digits classification. (I) Training accuracy and loss as a function of an epoch. (J) Confusion matrix for the classification test of the handwritten digits.

Non-volatile neuromorphic behavior can be realized by simple external circuit settings, such as connecting a switch (e.g. transistor) or a large resistor in series with the EP-IDNC device, to prevent unintentional self-discharging after programming. In our experiments, a 910 kΩ resistor is used. We first investigated the weight-updating property and state retention behavior of the non-volatile EP-IDNC device, recognizing their significance as pivotal neuromorphic parameters in the realization of high-performance hardware-based artificial neural networks (ANNs) designed for the implementation of AI algorithms. As shown in the long-term potentiation-depression (LTP-LTD) cycle curves, the EP-IDNC devices exhibit photonic weight (i.e. normalized transmittance change) exceeding 4 bits, and present very high linear weight update characteristics (Fig. 3A and B). The >4-bit weight is comparable and even greater than that of state-of-the-art photonic memory devices [48,63,64]. As one of the key parameters in neuromorphic devices, linearity has been further analyzed in detail from 30 repeated LTP-LTD cycles. At each programming step in LTP-LTD cycles, the weight updates value, i.e. normalized $\Delta T$, (Fig. S23) is extracted and analyzed using the cumulative distribution function (CDF), as shown in Equation 1.


(1) \begin{eqnarray*}
&&\quad\quad CDF\ \left( {Nor.\ \Delta T} \right)\nonumber\\
&&\quad\quad \quad= \mathop \smallint \limits_{ - 0.15}^{Nor.\ \Delta T} {p}_T\left( {Nor.\ \Delta T} \right)d\left( {Nor.\Delta T} \right),
\end{eqnarray*}

in which ${p}_T( {Nor.\ \Delta T} )$ is the probability distribution for $Nor.\ \Delta T$ (short for normalized $\Delta T$) under certain programming steps. As shown in the CDF heatmaps, the EP-IDNC devices maintain high weight updating linearity and excellent repeatability in 30 repeated LTP-LTD cycles (Fig. 3C and D). Interestingly, the weight of EP-IDNC devices can be programmed through voltage spikes or current spikes, and both can provide similar weight update characteristics with high linearity (Fig. S24), demonstrating the excellent neuromorphic behavior of our EP-IDNC devices. To measure the state retention behavior, we programmed the weight to a target value and fully disconnected the external circuit. The updated weight can maintain its value for more than 5 min (Fig. 3E). While the state retention time may not match that of inorganic memories, it aligns closely with state-of-the-art ECRAMs due to their similar working mechanisms [38,65]. Notably, our device, a redox-based electrochemical cell, fundamentally differs from the tiny capacitor-based design of dynamic random-access memory (DRAM), which requires refresh cycles. This distinction allows our device to be classified as a (quasi-)non-volatile memory, similar to ECRAMs when operated under open-circuit conditions, rather than a volatile memory. The 5-min state retention, although comparatively modest, proves ample for applications in online learning.

EP-IDNC devices’ non-volatile nature allows them to be used as convolutional kernels in a convolutional neural network (CNN) for extracting features from input data. As depicted in Fig. 3F, when the original image is projected onto a kernel composed of a non-volatile EP-IDNC device array, the transmitted light represents the element-wise product of the input light power and the device transmittance. Employing an optical fan-in, sum operations can be performed simultaneously to produce the processed image. Training algorithms can program the convolutional kernels, and the trained kernels can be stored in the EP-IDNC device array without requiring standby or inference power consumption. Reusing the stored kernels for different input images can significantly reduce the digital multiply-accumulate operations in CNNs. Figure 3G presents the simulation result (left) and experimental result (right) of the processed image. The left panel demonstrates that the kernel array is effectively trained to extract the original image's edge features. The experimental result confirms that the EP-IDNC device array is accurately programmed to the optimized value, thus effectively extracting edge features consistent with the simulation result.

To further illustrate the potential of using EP-IDNC devices in CNNs, a large-scale EP-IDNC-device-based CNN is simulated for a handwritten digital classification task (Fig. 3H). The network architecture comprises a convolutional layer with 32 3 × 3 kernels and a fully connected layer containing 32 input neurons and 10 output neurons, with all kernels implemented using the EP-IDNC device array. The CNN was trained on the MNIST dataset using a backpropagation algorithm, with the Adam optimizer set to a learning rate of 0.1. The network was trained over 20 epochs with a batch size of 512 to ensure convergence. Figure 3I displays the training accuracy and loss, demonstrating an accuracy of over 96.2%. The high accuracy underscores the crucial role of the EP-IDNC-based kernels in feature extraction. Figure S25A (accuracy vs. number of kernels) highlights their effectiveness, with performance scaling as the number of kernels increases. Furthermore, kernel performance is strongly influenced by the EP-IDNC device's modulation depth (Fig. S25B, accuracy vs. modulation depth). Notably, the modulation depth above 15 dB can be easily achieved by simply increasing the thickness of the p(g2T-T) electrochromic layer in EP-IDNC devices, as evidenced in Fig. S26. In addition, the modulation depth of EP-IDNC devices is much higher than existing photonic neuromorphic devices [44]. The confusion matrix for the test dataset (Fig. 3J) confirms the strong performance of the EP-IDNC-based CNN, with high accuracy across all digit classes when using devices with a 15-dB modulation depth.

### In-display neuromorphic computing

**Figure 4.**
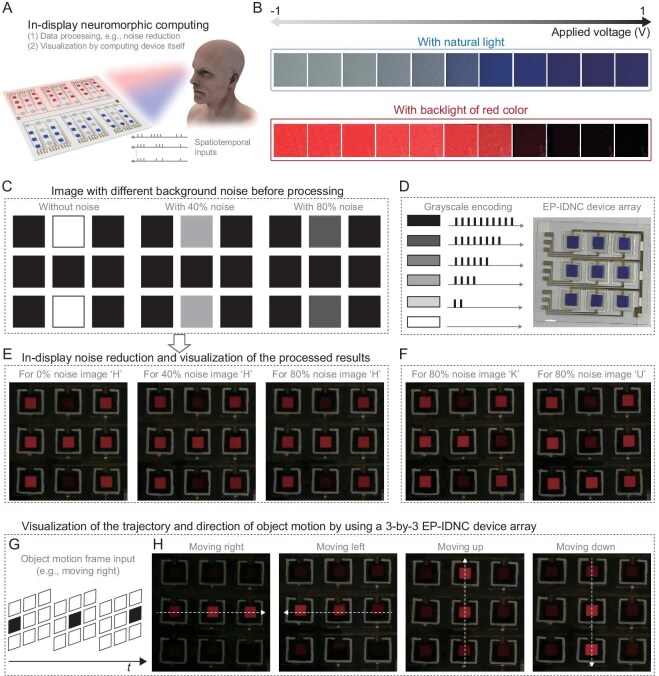
IDNC demonstration on a prototype 3 × 3 EP-IDNC devices array. (A) Schematic image shows the IDNC based on EP-IDNC devices. (B) Colors of the EP-IDNC device under different working voltages. The upper panel displays the color of the EP-IDNC device under natural light, and the lower panel displays the color of the EP-IDNC device with a red backlight applied. (C) Images with different background noise before being processed. (D) Grayscale encoding and the optical image of a prototype EP-IDNC array. (E and F) In-display noise reduction and visualization. (G) Schematic shows the static spatial frames of a moving object (i.e. moving right) as a function of time. (H) Visualization of the trajectory and direction of moving objects, including moving to the right, left, up and down.

Our EP-IDNC device has two methods to visualize the computing results: one relies on the natural color change of the electrochromic layer, and the other uses backlighting (Fig. 4A and B). The detailed implementation mechanisms, comparative advantages and limitations of these two methods in specific application scenarios are presented in Note S2. In the backlighting method, the EP-IDNC serves as a dynamic transmission backlight modulation device. The EP-IDNC device array can be seamlessly laminated on a flat, large-area light source, eliminating the need for individual light sources per pixel. Although both visualization methods can be employed, backlighting can better utilize the color modulation function of the EP-IDNC device (with the strongest modulation ability in the range of 600–650 nm), and achieve high visual contrast, and nighttime visualization of the computing results. Therefore, as a proof of concept, backlight-based visualization was used for demonstration.

We first conducted the real-time visualized image noise reduction based on our prototype 3 × 3 EP-IDNC array (Fig. 4C–F). Image pre-processing is one of the unique characteristics of the human eyes, playing a crucial role in improving the quality of sensory data. Images often contain a large amount of background noise. Large noise may completely bury the main features of the image (Fig. 4C). Neuromorphic devices with dynamic response and fading memory effect can function like our human retina to effectively suppress unnecessary noise and improve contrast, as well as highlight the main features of the image [4,66]. To demonstrate that our EP-IDNC array can process noisy images in real time and directly visualize the processing results, we use ‘grayscale encoding’ to convert the pixel grayscale of the image into the number of voltage pulses and apply it to the array. To make sure all the pixels of the EP-IDNC array are black in the initial state (i.e. no red light passing through pixels), the pulse baseline was set at 1 V. Resistors of 4.3 kΩ were intentionally connected with the voltage control terminal of each pixel of the array to control the charging and discharging rate so that the color change of each pixel can be visualized obviously. The main feature with a grayscale value of ‘100%’ is encoded into 10 pulses (from 1 to −2 V, with a duty cycle of 60%), while the number of encoded pulses for background noise is determined by the size of the noise, e.g. eight pulses for 80% background noise. Due to the STM effect of EP-IDNC, feature information will gradually increase until all 10 pulses are applied, while noise information, although initially enhanced, will begin to dissipate after the corresponding pulse of noise ends. All noise reduction processes are real-time visualizable by our eyes and do not require complex readout systems. For the image with the letter ‘H’, even if the background noise reaches over 80%, our device can still perform effective noise reduction and visualize the same output result as that of an image without noise (Fig. 4E). Moreover, the visual noise reduction and the IDNC abilities of our array are also applicable to other images with high background noise, e.g. images with letters ‘K’ and ‘U’ (Fig. 4F).

Next, we demonstrated another IDNC application, which utilizes an EP-IDNC array to process and visualize motion objects (Fig. 4G and H). Traditionally, moving objects can be recorded by sensors, but their output information is static spatial frames. Time-related information is often not integrated into the spatial frames. The expression of motion information typically requires a large amount of spatial frame data. In addition, it has been proven that motion processing is a computationally challenging task and requires considerable computational resources. Fusing spatiotemporal information into one frame could be an intriguing way to process motion-related data [67]. In our demonstration, the static spatial frames of a moving object as a function of time have been further converted into voltage pulse streams applied to the EP-IDNC array (Fig. 4G). By utilizing the STM and visualization capabilities of an EP-IDNC array, spatiotemporal motion information can be integrated through the temporal variation of the brightness of each pixel, as pixels that receive stimulus information earlier have lower brightness due to their dynamic fading characteristics, while devices that ultimately receive stimuli will exhibit higher brightness. As shown in Fig. 4H, the trajectory contour of the entire motion from left to right, from right to left, from bottom to top, and from top to bottom was successfully processed and displayed using the EP-IDNC array. At the current stage, motion perception and visualization were demonstrated by using a very simple object, and assuming it moved pixel by pixel without pixel overlapping. In a real application, the moving object will be a complex image and its movement will cause pixel overlapping. In that case, the overlapped pixels will be brighter as they receive more stimuli. As time goes by, the image with different brightnesses of each pixel will appear on the array to show the spatiotemporal information of the moving objects. Therefore, our EP-IDNC array is also expected to be applicable in complex object movement even though we haven't done this demonstration due to the limited device size and experimental conditions of our lab at the current stage. The primary technical bottleneck lies in the sub-optimal patterning process of Ag/AgCl electrodes, where manual drawing produces electrodes with widths of approximately 1 mm. This limitation fundamentally restricts device miniaturization and high-density integration. To scale this technology for practical applications, it is essential to adopt advanced manufacturing techniques such as inkjet printing or photolithography to achieve high-resolution patterning of Ag/AgCl electrodes. These methods could achieve feature sizes of less than 100 μm, enabling large-scale array production. These engineering advancements would not only enhance complex motion analysis capabilities but also pave the way for practical neuromorphic visualization systems.

**Figure 5.**
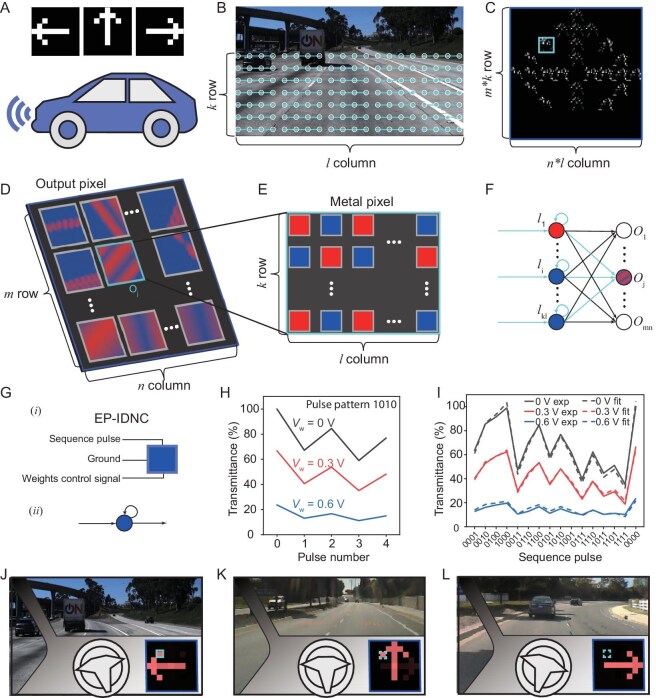
IDNC system for car steering reminder. (A) Schematic images show the visualized steering reminder using the triple-terminal EP-IDNC device-based in-display computing system. (B) Road lane image overlaid with sensor data, split into an array of pixels represented by *k*-row and *l*-column. (C) Weight image after training the in-display computing system, showing pixel patterns generated from the EP-IDNC array. The cyan box highlights the *k*-row and *l*-column matrix values corresponding to the output meta-pixel *O*_j_. (D and E) Schematic of the output of the 2-terminal EP-IDNC device array, which forms *m*-row and *n*-column of output meta-pixels. The cyan box highlights an output meta-pixel *O*_j_. Each meta-pixel is formed by *k*-row and *l*-column 2-terminal-EPCN devices taking the *k* × *l* sequence input as shown in (B), and weighted by *k* × *l* weight signal, as shown in (C). Such a meta-pixel physically implements an output node of a reservoir computing, as shown in (F). (G) Schematic of the (*i*) triple-terminal EP-IDNC device with a sequence pulse input, ground and weight control signal (*V*_w_) and (*ii*) the simplified representation of the device functionality. (H) Transmittance responses of the EP-IDNC device under different *V*_w_, tested using a sequence pulse pattern ‘1010’. (I) Experimental and fitted transmittance results for different sequence pulse patterns and *V*_w_. The results indicate distinct trends in transmittance behavior based on the applied *V*_w_. (J–L) Simulated results for the car steering reminder task, showing the visual output in real time based on road lane data and EP-IDNC device computations. Arrows indicating the direction of the turn are generated and displayed on the driver interface.

To further illustrate the potential of our EP-IDNC device and the concept of IDNC proposed in this work, we further conducted a large-scale simulation for a visualized copilot system (Fig. 5, Fig. S27 and Note S3). This system processes road lane information captured by vehicle sensors and generates a visualized steering reminder for the driver. The road lane image is first divided into an array of *k*-row and *l*-column pixels (Fig. 5B) where each pixel's sequence data is processed by the EP-IDNC devices. To properly process the sequence data, the trained weights are represented as a *k* × *l* matrix of weight values (Fig. 5C). These weights are computed using backpropagation. The cyan box in Fig. 5C highlights the weight matrix that corresponds to the output meta-pixel *O*_j_, which is part of the larger meta-pixel grid formed by the EP-IDNC devices. Each meta-pixel is composed of *k* × *l* EP-IDNC devices, which process the input sequence to generate an output, representing an element in the steering reminder. The layout of the output meta-pixels is schematically shown in Fig. 5D, where the cyan box highlights a specific output meta-pixel *O*_j_. Each meta-pixel physically implements an output node in the reservoir computing framework (Fig. 5F). Next, we zoom into the structure of the meta-pixel. As shown in Fig. 5E, each meta-pixel is composed of smaller elements arranged in a *k*-row × *l*-column configuration. These sub-elements receive the sequence pulses and weight control signals to process the data from the road image.

The triple-terminal EP-IDNC devices within each meta-pixel are responsible for processing sequence input pulses and control weights simultaneously, as depicted in Fig. 5G and Fig. 1C. Specifically, one terminal of the triple-terminal EP-IDNC receives sequence pulses, the second terminal receives a weight control signal for weight modulation, while the third terminal is grounded. The transmittance of the triple-terminal EP-IDNC is tested after exposure to different sequence pulses under varying weight control signals, as depicted in Fig. 5H and I, and Fig. S28. To accurately capture the unique features of our triple-terminal EP-IDNC, we employ the following equation to merge the effects of the weight control signal and sequence pulse:


(2) \begin{equation*}
T = - 1.569 + 1.589\times{e}^{\left( {{T}_{\mathrm{weight}}\times{T}_{\mathrm{sequence}}\times{0.5}} \right)},
\end{equation*}

where *T*_weight_ is the transmittance obtained by only applying the weight control signal (i.e. sequence pulse is set to 0000), and the *T*_sequence_ is the transmittance obtained by applying sequence pulses while setting the weight control signal at zero. *T*_weight_ and *T*_sequence_ are experimentally measured in Fig. 5I. The simulated results generated by Equation 2 align closely with the experimental results. Thus, the device characteristics utilized in the IDRC simulation are guided by Equation 2.

The simulated results for the car steering reminder task are shown in Fig. 5J–L and Video S1, where arrows are displayed on the driver's interface, indicating whether to turn left, right or go straight on, based on the real-time data processed by our IDNC system.

## DISCUSSION

By harnessing the electrochromism of OSCs, we have developed an innovative prototype of an all-in-one EP-IDNC device tailored for IDNC applications. This unique electrochromism empowers the EP-IDNC device with customizable synaptic plasticity for information encoding and in-memory processing, along with the ability to visually represent computing results through device color changes. Moreover, the device exhibits excellent endurance (>1000 switching cycles) and long-term storage stability (>1 month), confirming its practical reliability (Figs S29 and S30). While the photonic reading operation enables ultra-low inference energy consumption, the current programming energy remains higher than that of state-of-the-art neuromorphic devices due to the relatively large device size—a challenge addressable through future miniaturization efforts. Using a 3 × 3 EP-IDNC array, we successfully demonstrated key IDNC functionalities, including noise reduction and motion object perception. Simulations further reveal the system-level potential of our EP-IDNC devices, showcasing multi-terminal operation, multi-level weight updating, spatiotemporal information processing, and color-based visualization, as exemplified by applications like a car steering reminder. Our work not only establishes the feasibility of electrochromic-material-based IDNC but also introduces the broader concept of ‘in-display neuromorphic computing,’ opening new avenues for next-generation interactive neuromorphic systems.

## MATERIALS AND METHODS

### Materials

All processing solvents, including chloroform and ethanol, were acquired from Sigma–Aldrich and utilized without further purification. 1-Ethyl-3-methylimidazolium bis(trifluoromethylsulfonyl)imide ([EMIM]^+^[TFSI]^−^ (purity ≥ 99%), α,ω-diacrylated poly(ethylene glycol) (PEG-DA) oligomer (number average molecular weight, Mn = 575), and 2-hydroxy-2-methylpropiophenone (HOMPP) (purity ≥ 98%) were all sourced from Sigma–Aldrich and utilized without additional treatment. The conjugated polymer semiconductor poly(2-(3,3′-bis(2-(2-(2-methoxyethoxy)ethoxy)ethoxy)-[2,2′-bithiophen]-5)yl thiophene) (p(g2T-T)) was synthesized using previously reported methods [53]. The azide-based crosslinker 2,2-bis(((4-azido2,3,5,6-tetrafluoroben-zoyl)-oxy)methyl)propane1,3-diylbis(4-azido-2,3,5,6 tetrafluorobenzoate) (4Bx) was synthesized following a procedure reported in the literature [68]. The patterned ITO electrodes on PET substrates were obtained from Shenzhen South China Xiangcheng Technology Co., Ltd. The Ag/AgCl paste was purchased from Shanghai Julong Technology Co., Ltd.

### Fabrication of the EP-IDNC device and array

The p(g2T-T) was dissolved in chloroform (5 mg/mL). Before usage, the solution underwent stirring under nitrogen protection at 60°C for a minimum of 12 h. For the manufacturing of EP-IDNC devices, the p(g2T-T) solution was spin-coated onto the surface of an ITO-PET film. Unwanted areas were then removed using cotton swabs containing ethanol to form semiconductor patterns. Alternatively, patterning of the p(g2T-T) film could be achieved using photo-crosslinking agent (4Bx)-enabled photopatterning [25]. The Ag/AgCl electrode was formed by drawing the Ag/AgCl paste with a pen. Subsequently, the device was heated at 100°C for 1 h to eliminate the solvent in the semiconductor and the Ag/AgCl electrode. A mixed solution containing [EMIM]^+^[TFSI]^−^, PEG-DA and HOMPP (88:8:4, w/w) was drop-coated onto the patterned p(g2T-T) film and Ag/AgCl electrode and then flattened using a clear PET sheet. The device was then placed in a UV curing machine and subjected to 1 min of UV irradiation. Multi-terminal EP-IDNC devices and device arrays were fabricated using the same methods with different ITO electrode patterns. To achieve a thick p(g2T-T) film, an azide-based photo-crosslinker, 4Bx (5% w/w), was blended with the p(g2T-T) solution. Multiple rounds of spin-coating and UV crosslinking were employed. The resulting crosslinked film exhibited high resistance to chemical solvents, enabling the increase in thickness of the p(g2T-T) layer through repeated spin-coating and UV irradiation processes.

### Characterization and measurement

UV-vis of the p(g2T-T) films was conducted using a spectrometer (Lambda 950, PE Inc., USA). X-ray photoelectron spectroscopy (XPS) analysis was carried out using an ESCALAB 250Xi instrument. Monochromatic light was provided by a xenon lamp source equipped with a dual grating monochromator (Omno 330150, Beijing NBeT, China). The performance of the EP-IDNC device was evaluated using power and energy meter consoles and interfaces (PM100D, Thorlabs) along with compatible optical power monitor software. Programming voltage was applied using an arbitrary waveform generator (Rigol DG4162). Basic device performance testing was conducted in a homemade dark box.

## Supplementary Material

nwaf224_Supplemental_Files
