# Supplementary material for: Electrically programmable organic in-display neuromorphic computing
Source: Natl Sci Rev. 2025 Jun 4;12(8):nwaf224. doi: 10.1093/nsr/nwaf224 (PMC12260503; doi:10.1093/nsr/nwaf224)
Supplement: nwaf224_Supplemental_Files [file nwaf224_supplemental_files.zip › Supplementary data.pdf]

Supporting Information for

Electrically programmable organic in-display neuromorphic computing

Shilei Dai[a,†], Dingchen Wang[a,b,†], Xu Liu[c,†], Binbin Cui[a], Xinyu Tian[a], Dingyao Liu[a], Huawei Hu[d], Pu Guo[c], Tongrui Sun[c], Junyao Zhang[c], Jia Huang[c,]*, Shiming Zhang[a,]* and Zhongrui Wang[a,b,]*

[a]Department of Electrical and Electronic Engineering, The University of Hong Kong, Hong Kong 999077, China;

[b]School of Microelectronics, Southern University of Science and Technology, Shenzhen 518055, China;

[c]School of Materials Science and Engineering, Tongji University, Shanghai 201804, China;

[d]State Key Laboratory for Modification of Chemical Fibers and Polymer Materials & College of Materials Science and Engineering, Donghua University, Shanghai 201620, China

***Corresponding authors**. E-mails: huangjia@tongji.edu.cn; szhang@eee.hku.hk; zrwang@eee.hku.hk

[†]Equally contributed to this work.

This PDF file includes:

Notes S1-3
Figures S1 to S30
Tables S1
Legends for Movies S1
SI References

Other supporting materials for this manuscript include the following:

Movies S1

Note S1. Precise discharge rate and memory retention control mechanism.

The EP-IDNC device operates similarly to a redox-based electrochemical cell, with the p(g2T-T) electrochromic layer serving as the working electrode and the Ag/AgCl electrode acting as the reference electrode. Following programming operations, the open circuit voltage, i.e., $V_{oc}(t)$, between the p(g2T-T) electrochromic layer and Ag/AgCl will spontaneously drive the charges back to their initial equilibrium state. The discharge kinetics can be mathematically described by:

$$V_{oc}(t) = \frac{1}{C}\left(Q_0 - \int_0^t \frac{V_{oc}(t)}{R} dt\right) \tag{1}$$

Where t is the time after programming, Q_0 is the number of charges stored in the p(g2T-T) electrochromic layer after programming, C is the capacitance of the p(g2T-T) electrochromic layer, and R is the value of the regulation resistance. Through deliberate modulation of R, we achieve deterministic control over the RC time constant ($\tau = RC$), thereby enabling precise tuning of the discharge rate.

Equation 1 reveals two working modes:

- *(Quasi-) Non-volatile mode*: Implemented through either: (i) a physical switch to disconnect the external circuits immediately after programming or (ii) using a large R that can prevent quick discharging so that quasi-non-volatile characteristics can be realized.
- *Volatile mode*: Implemented through using low R values to facilitating rapid discharging.

Note S2. **Comparison between nature color change and backlighting methods used in EP-IDNC devices.**

➢ **<u>For natural color changes</u>**

(1) Implementation Mechanism: The device's natural color transitions originate from the electrochromic properties of the p(g2T-T) OMIEC film. Specifically, reversible electrochemical doping/de-doping processes modulate the film's optical absorption spectrum, enabling visible color shifts (e.g., transparent ↔ opaque states) in response to applied pulse patterns. This intrinsic color modulation directly reflects computing outputs without requiring external light sources.

(2) Advantages and Limitations

- **Strengths:**
  - *(i) Low-Power Operation*: This relies solely on electrochemical doping, eliminating the need for active backlight components.
  - *(ii) Ambient Light Compatibility*: Delivers high-contrast visualization under bright illumination (e.g., daylight), leveraging ambient light to enhance visibility.

- **Challenges:**
  - *(i) Low-Light Limitations*: In dim environments, the absence of emissive properties reduces visibility and contrast, restricting use cases to well-lit settings.
  - *(ii) Dynamic Range*: Color saturation may vary depending on film thickness.

➢ **<u>For backlighting</u>**

(1) Implementation mechanism: The device operates as a dynamic transmission modulator when paired with a backlight unit. The p(g2T-T) film's electrochromic state controls the transmittance of an external light source (e.g., LED), enabling programmable output colors by filtering specific wavelengths.

(2) Advantages and Limitations:

- **Strengths:**
  - *(iii) Universal Visibility*: Functions effectively in both bright and dim environments due to the active backlight, ensuring consistent contrast regardless of ambient conditions.
  - *(iv) Color Flexibility*: Supports a broader color gamut by combining backlight wavelengths with film modulation.

- **Challenges:**
  - *(v) Increased Power Consumption:* Backlight integration raises overall energy demands, limiting suitability for battery-dependent applications.
  - *(vi) System Complexity:* Future integration requires embedding a light source, complicating device miniaturization, and raising manufacturing costs.

- **Scenario-Driven Recommendations**

(1) Natural Color Mode is optimal for energy-constrained applications in well-lit environments (e.g., outdoor IoT sensors, daylight-readable displays).

(2) Backlighting Mode suits scenarios requiring visibility in variable lighting (e.g., indoor human-machine interfaces, medical displays), albeit with trade-offs in power efficiency and design simplicity.

Note S3. Integrated in-display computing system simulation

The IDNC system utilizes a triple-terminal EP-IDNC device array. Each EP-IDNC device receives two critical inputs: a sequence pulse and a weight control signal. The input road lane image is divided into a 12-row by 48-column grid, with every 4 pixels forming a sequence, resulting in a 12-by-12 input sequence matrix. These sequences are then transformed into temporal electrical pulses based on the pixel intensity of the image. White pixels, representing the road lane, are assigned low voltage ('0' signal), while dark blue pixels, corresponding to non-lane areas, are assigned high voltage. These sequence pulses are distributed 9 by 9 times across the EP-IDNC array. Each EP-IDNC device operates under a weight control signal, which is pre-determined via backpropagation training. The overall array, composed of 108 rows and 108 columns of EP-IDNC devices, is organized into meta-pixels. Each meta-pixel processes specific sequence inputs along with the corresponding weight control signal. The total transmittance from each meta-pixel is summed to generate an output. This process is repeated across the entire array to produce the final steering reminder. To understand the behavior of the triple-terminal EP-IDNC device, we first examine its transmittance function, which is influenced by the interaction of the sequence pulse and weight control signal. When the weight control voltage is set to zero, the device transmittance depends solely on the sequence pulses, referred to as T_{sequence}. Conversely, when the sequence pulses are set to '0000,' the transmittance, labeled as $T_{\text{weight,}}$ is determined by the weight control voltage. Both T_{sequence} and T_{weight} range between 0 and 1. We employ equation 2 to accurately capture the unique features of our triple-terminal EP-IDNC. The values of -1.569 and 1.589 were obtained by performing a nonlinear least-squares fit to the experimental data in **Fig. S28** using the scipy.optimize module in Python, minimizing the discrepancy between the experimental data and the fitted curves. The fitting quality was rigorously validated by a coefficient of determination (R^2) of 0.9936, a mean absolute percentage error (MAPE) of 6.10%, and a residual sum of squares (RSS) of 0.0206, confirming the statistical reliability of the parameters. This close fit to the experimental data enables accurate simulation of the device's transmittance under different conditions. We then implement this transmittance model in the IDNC system by customizing an RC framework using the Keras platform. In this modified model, the standard multiplication operation is replaced with the triple-terminal EP-IDNC's transmittance function. The T_{sequence} for each device is calculated by mapping the segmented road lane sequence data to the measured transmittance under zero weight control voltage. Optimization of T_{weight} for each device is performed using the backpropagation algorithm, fine-tuning the system to process the input data effectively. This optimized T_{weight} is crucial for generating accurate steering reminders. Simulation results demonstrate that this system can process road data in real time, providing a user-friendly, visualized steering reminder that can assist drivers with navigation. The integration of the triple-terminal EP-IDNC transmittance model into the reservoir computing framework allows for high-speed, efficient processing of visual data, confirming the viability of this IDNC approach.

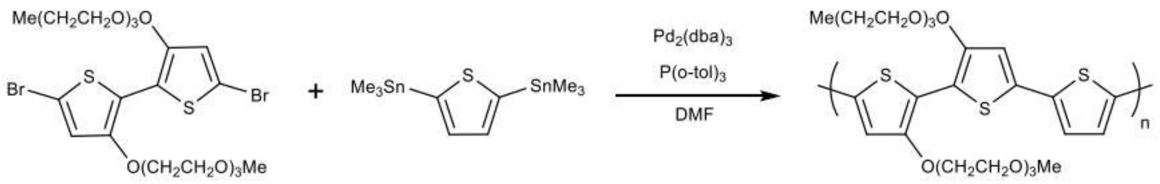

Fig. S1. The synthesis procedure of p(g2T-T). The p(g2T-T) is synthesized according to the literature reports [1].

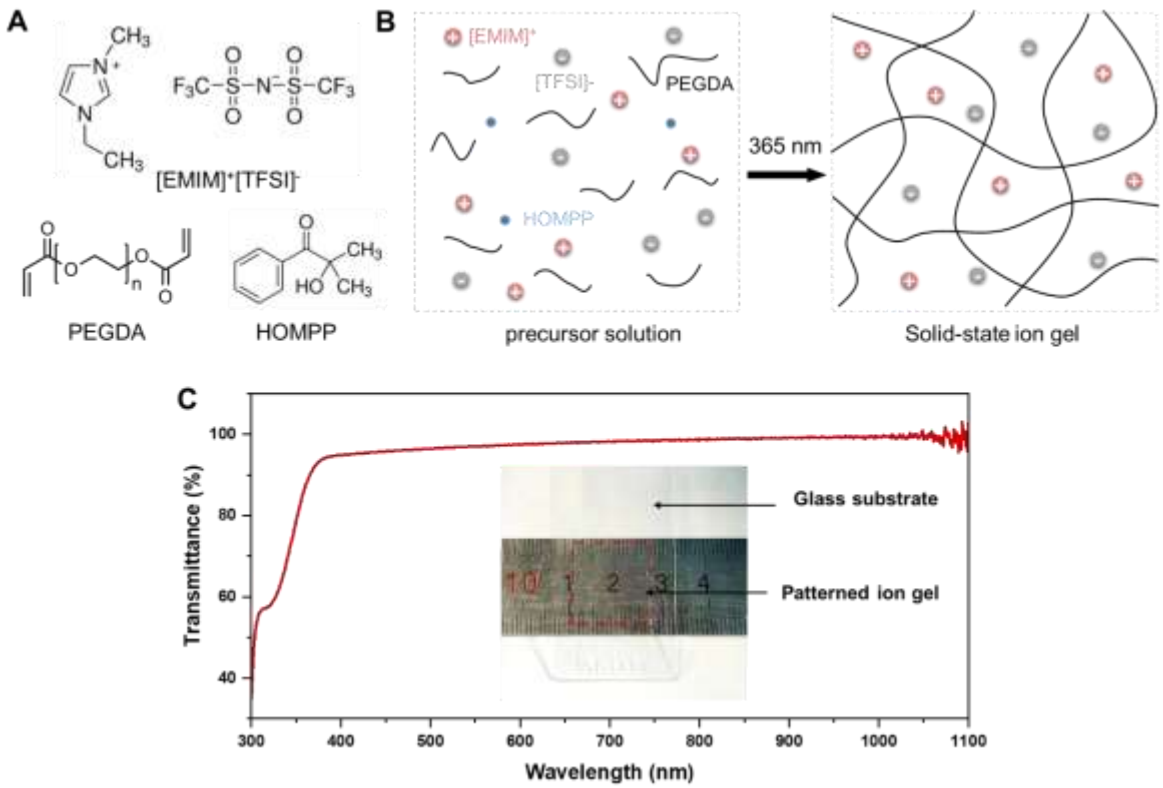

Fig. S2. Photo-patternable ion gel. (A) Molecular structures of the ionic liquid ($[EMIM]^+[TFSI]^-$), PEGDA oligomers and the photo-initiator (HOMPP) for the preparation of transparent solid-state ion gel. **(B)** Photo-patternable process of the ion gel. **(C)** Transmittance of the photopatterned ion gel. The insert image shows the photopatterned ion gel on glass substrate.

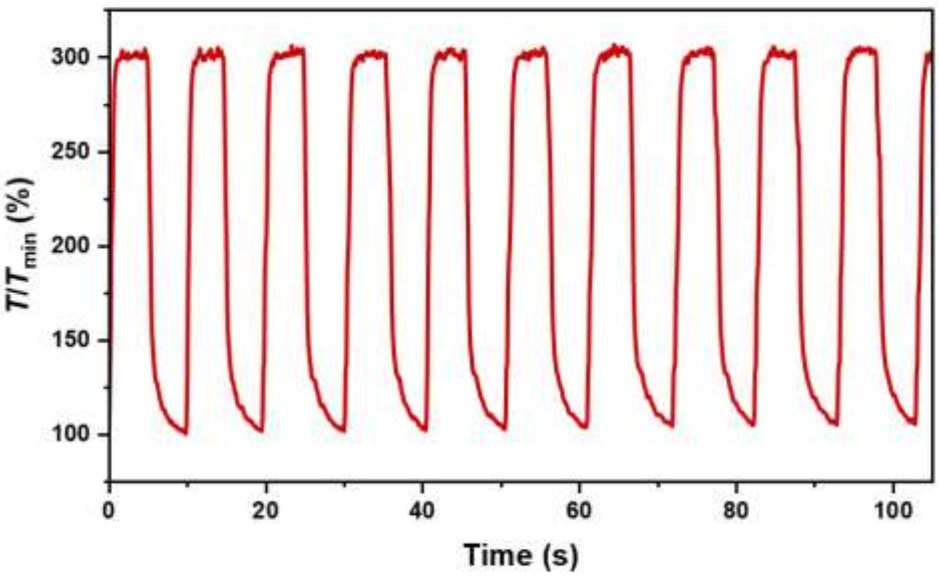

Fig. S3. Switching contrast. The switching contrast is measured by applying square voltage pulses (1 to -1 V) to the EPNC device. A high switching contrast (>300%) is obtained.

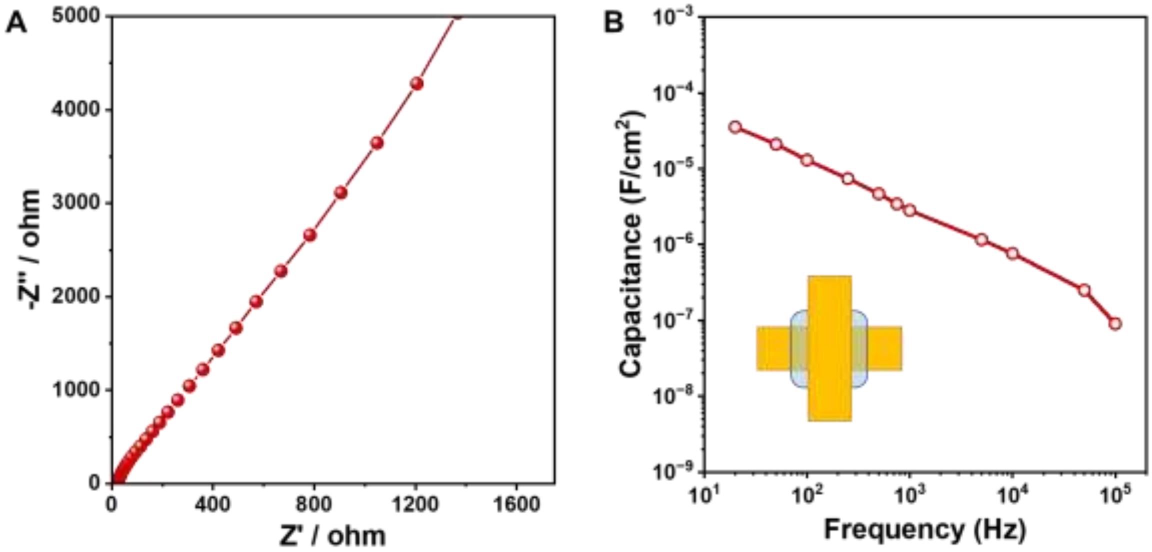

Fig. S4. Ion gel performance. (**A**) Electrochemical impedance spectroscopy (EIS) and (**B**) capacitance measurement of photopatterned ion gel.

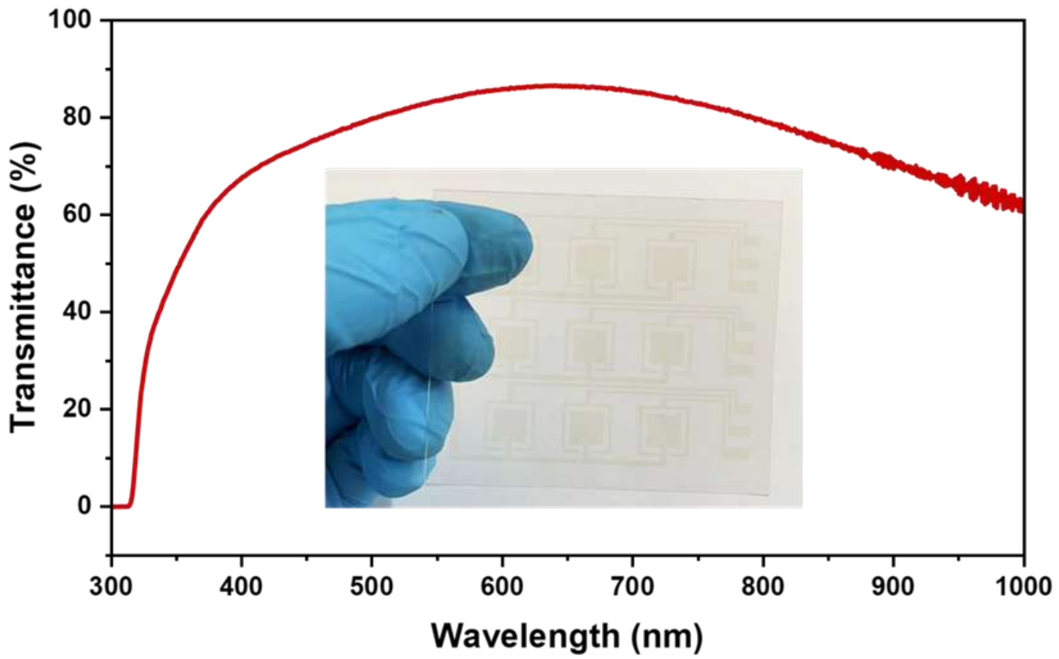

Fig. S5. Transmittance of the ITO-PET electrode array. The insert shows the optical image of the ITO electrode array on PET substrate. The transmittance was measured on the area where both ITO and PET exist.

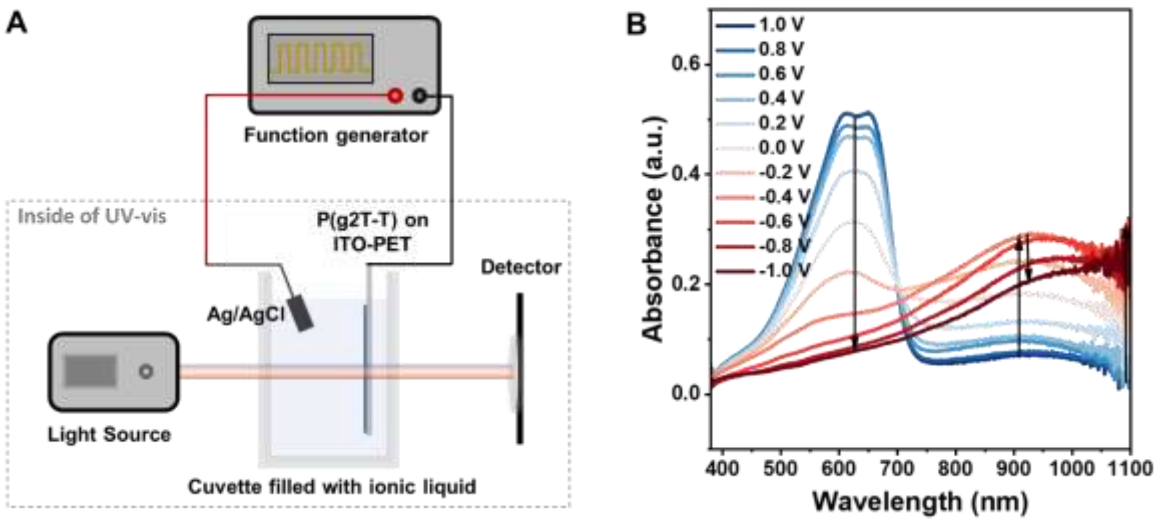

Fig. S6. UV-vis absorption spectra of p(g2T-T). (**A**) Schematic diagram of the optical measurement setup. (**B**) UV-vis of P(g2T-T) at different control voltage.

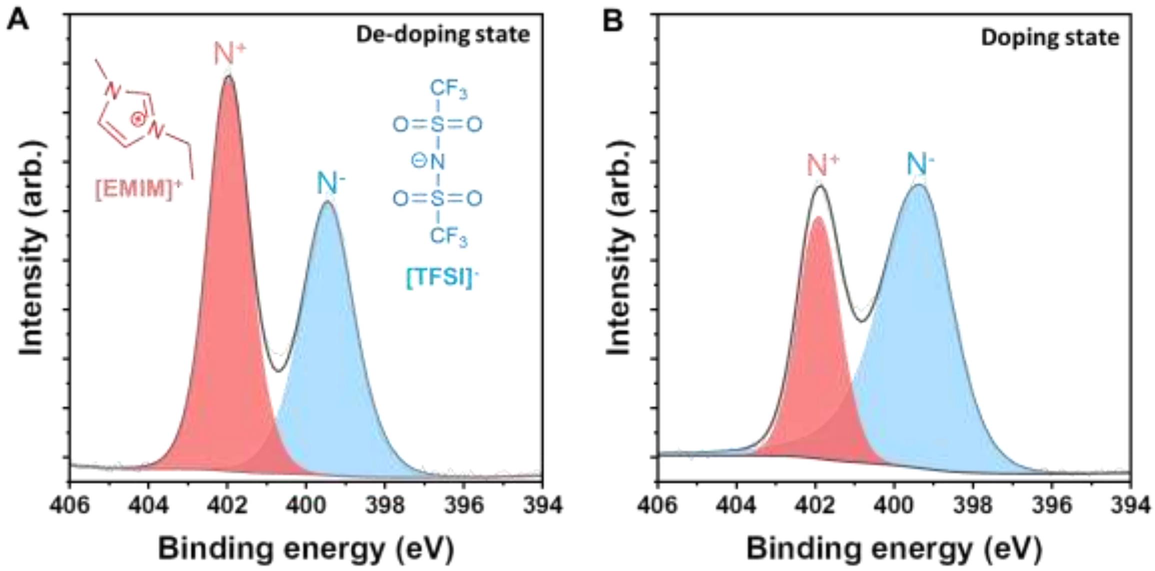

Fig. S7. XPS measurement. (**A**) Measured after applying a voltage of -0.8 V *vs*. Ag/AgCl electrode. (**B**) Measured after applying a voltage of 0.8 V *vs.* Ag/AgCl electrode.

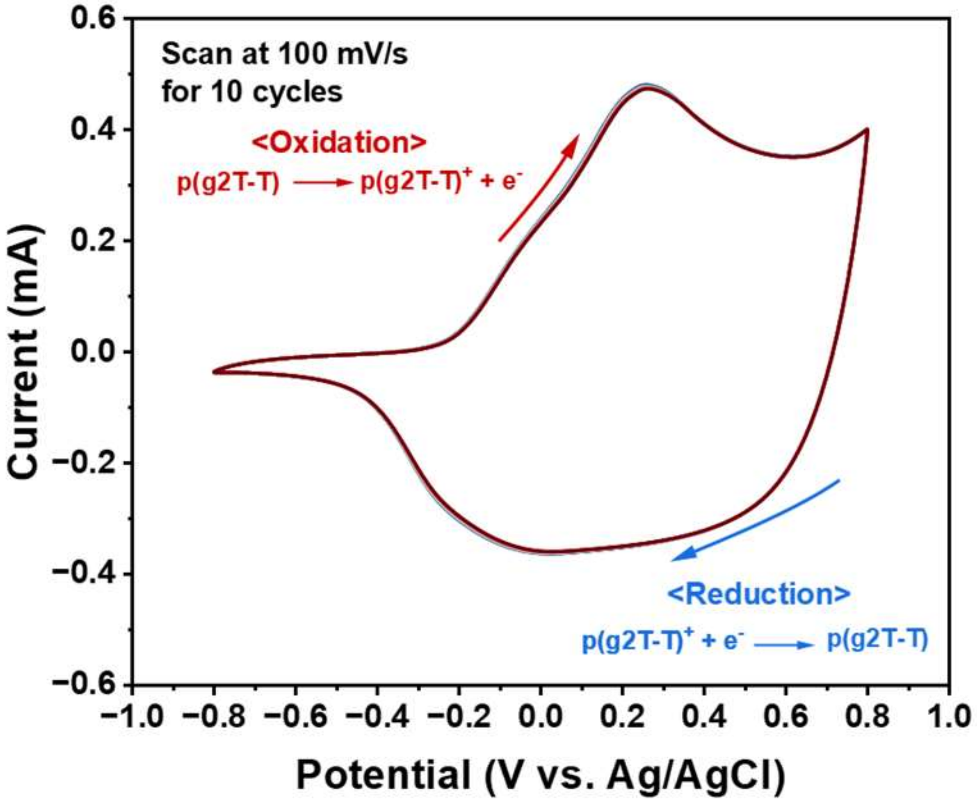

Fig. S8. Cyclic voltammogram (CV) curve of p(g2T-T) in [EMIM]$^+$[TFSI]$^-$ ionic liquid. The CV curve was tested at 100 mV/s for 10 times to evaluate cyclic stability and revisability of p(g2T-T).

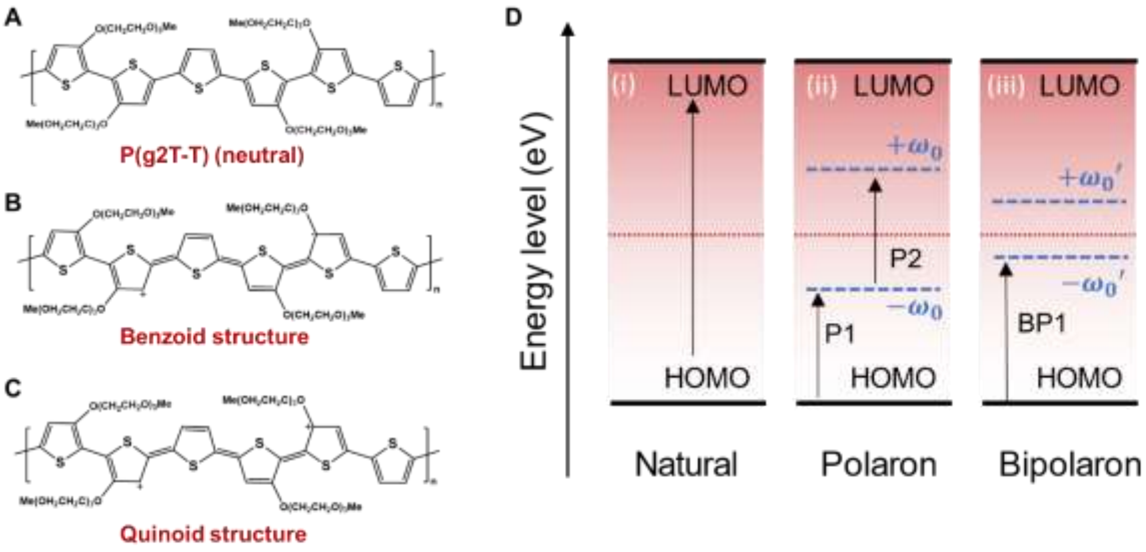

Fig. S9. Molecular structure of P(g2T-T). (**A**) Neutral state; (**B**) A positive polaron (Benzoid structure); and (**C**) A positive bipolaron (Quinoid structure). (**D**) Schematic energy level of p(g2T-T) film at different doping states.

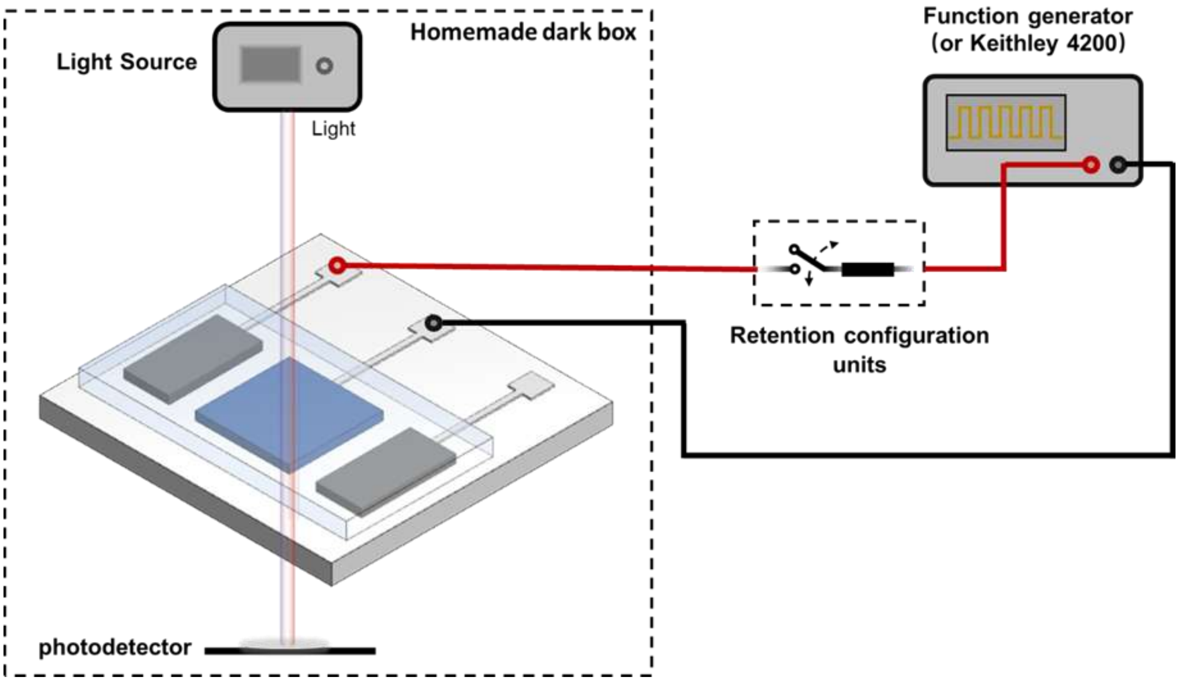

Fig. S10. Diagram of the experimental setup. The operation mode/or state retention time can be configured by using the retention configuration units, i.e., resistors or switches.

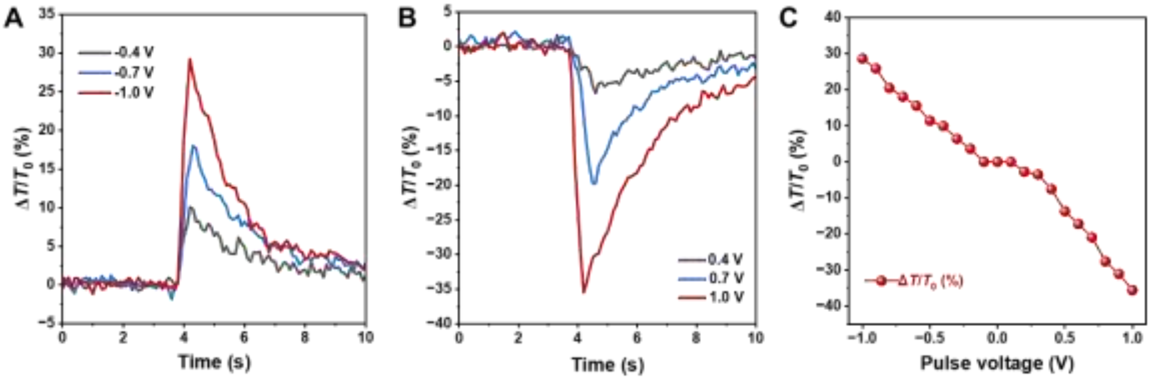

Fig. S11. Pulse intensity modulated EPSP/IPSP. (**A**) EPSPs; (**B**) IPSPs; (**C**) Peak EPSP/IPSP value as a function of pulse voltage.

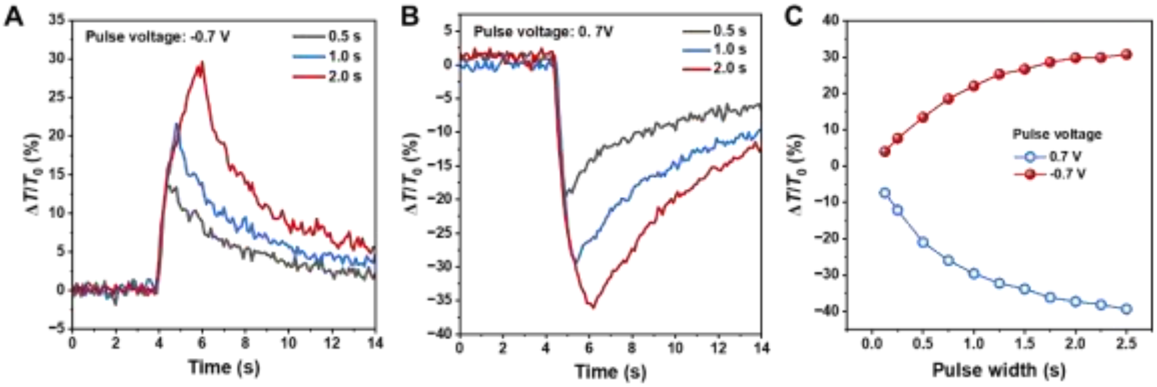

Fig. S12. Pulse width modulated EPSP/IPSP response. (**A**) EPSPs; (**B**) IPSPs; (**C**) Peak EPSP/IPSP value as a function of pulse width.

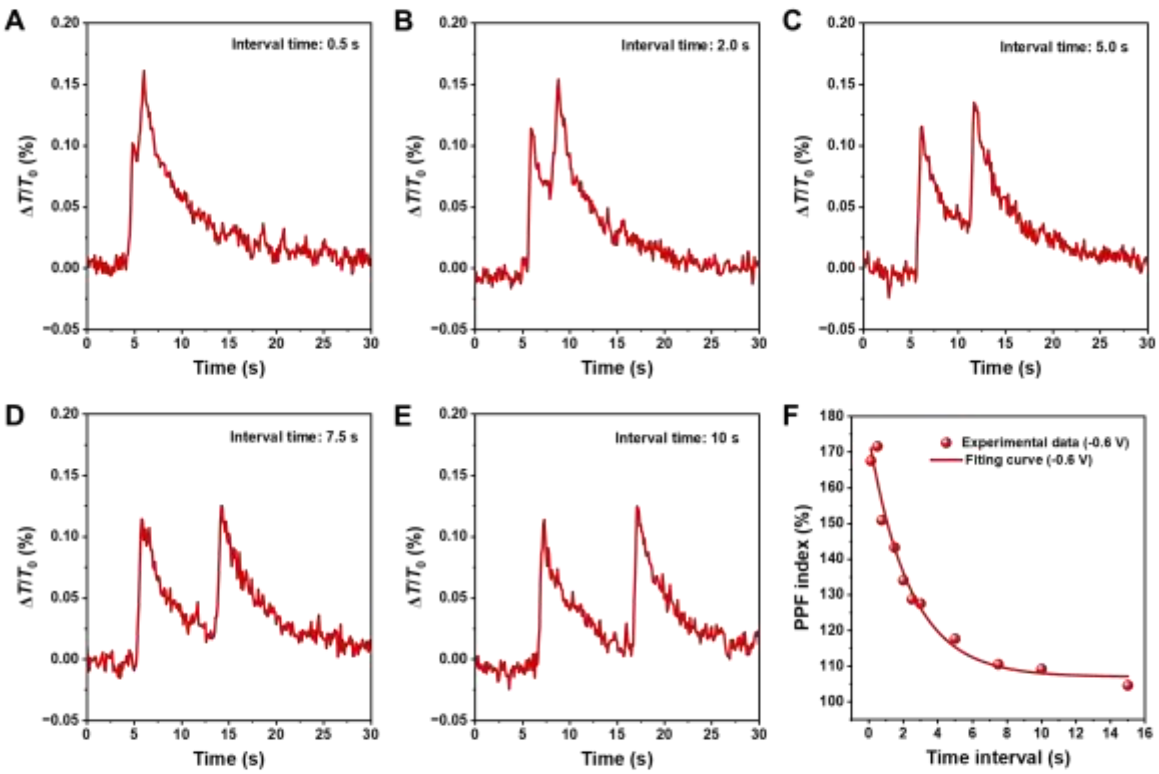

Fig. S13. PPF index. (**A-E**) EPSPs of the EP-IDNC device triggered by two consecutive pulses at different time intervals; (**F**) PPF index as a function of time interval of two consecutive pulses.

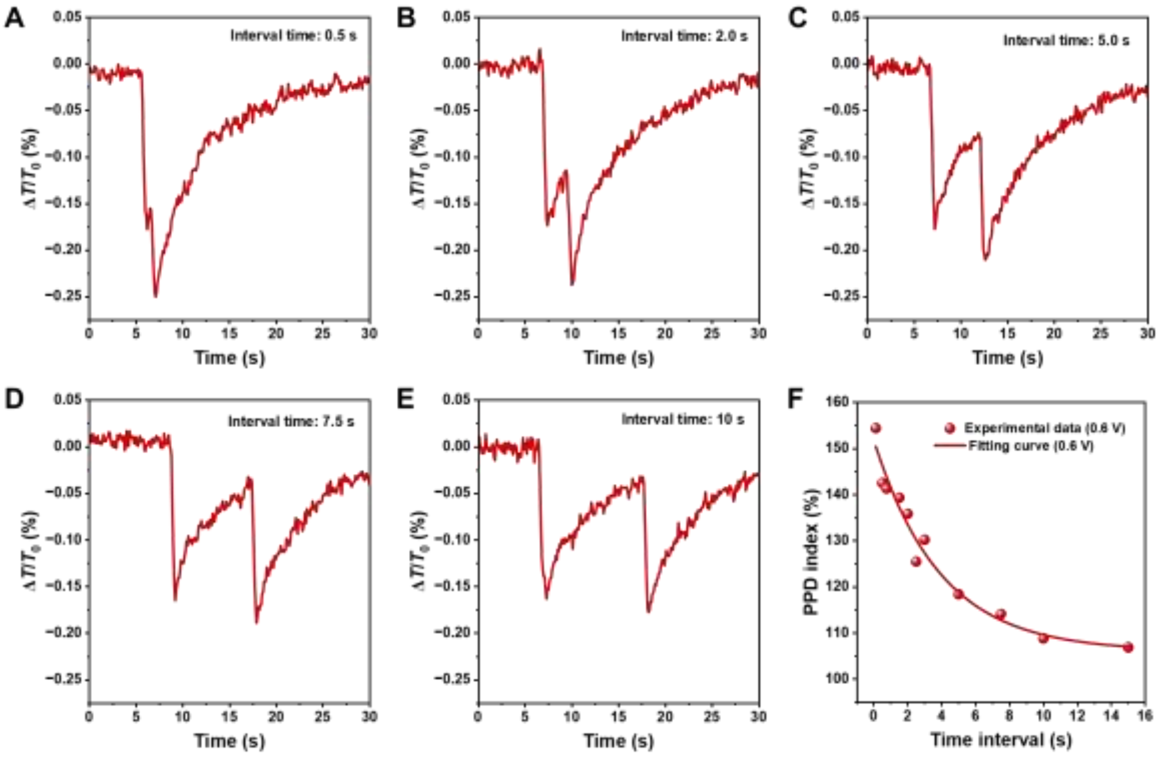

Fig. S14. PPD index. (**A-E**) IPSPs of the EP-IDNC device triggered by two consecutive pulses at different time intervals; (**F**) PPD index as a function of time interval of two consecutive pulses.

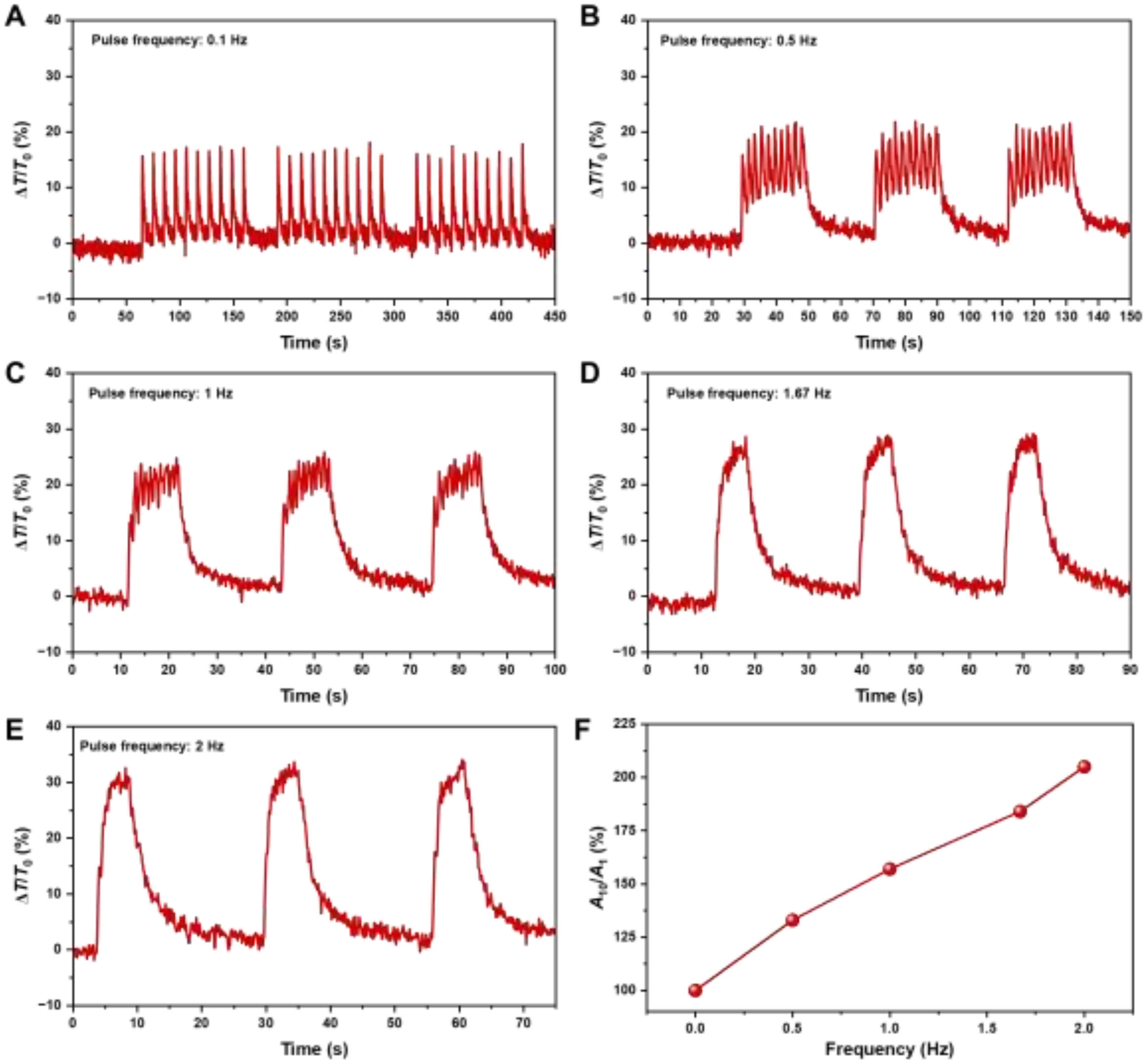

Fig. S15. Spike-rate dependent plasticity. (**A-E**) EPSPs of the EP-IDNC device triggered by 10 consecutive pulses at (A) 0.1 Hz, (B) 0.5 Hz, (C) 1 Hz, (D) 1.67 Hz, and (E) 2 Hz. Each pulse trains were repeated for 3 times. (**F**) The ratio of the 10th pulse peak response to the 1th pulse peak response as a function of pulse frequency.

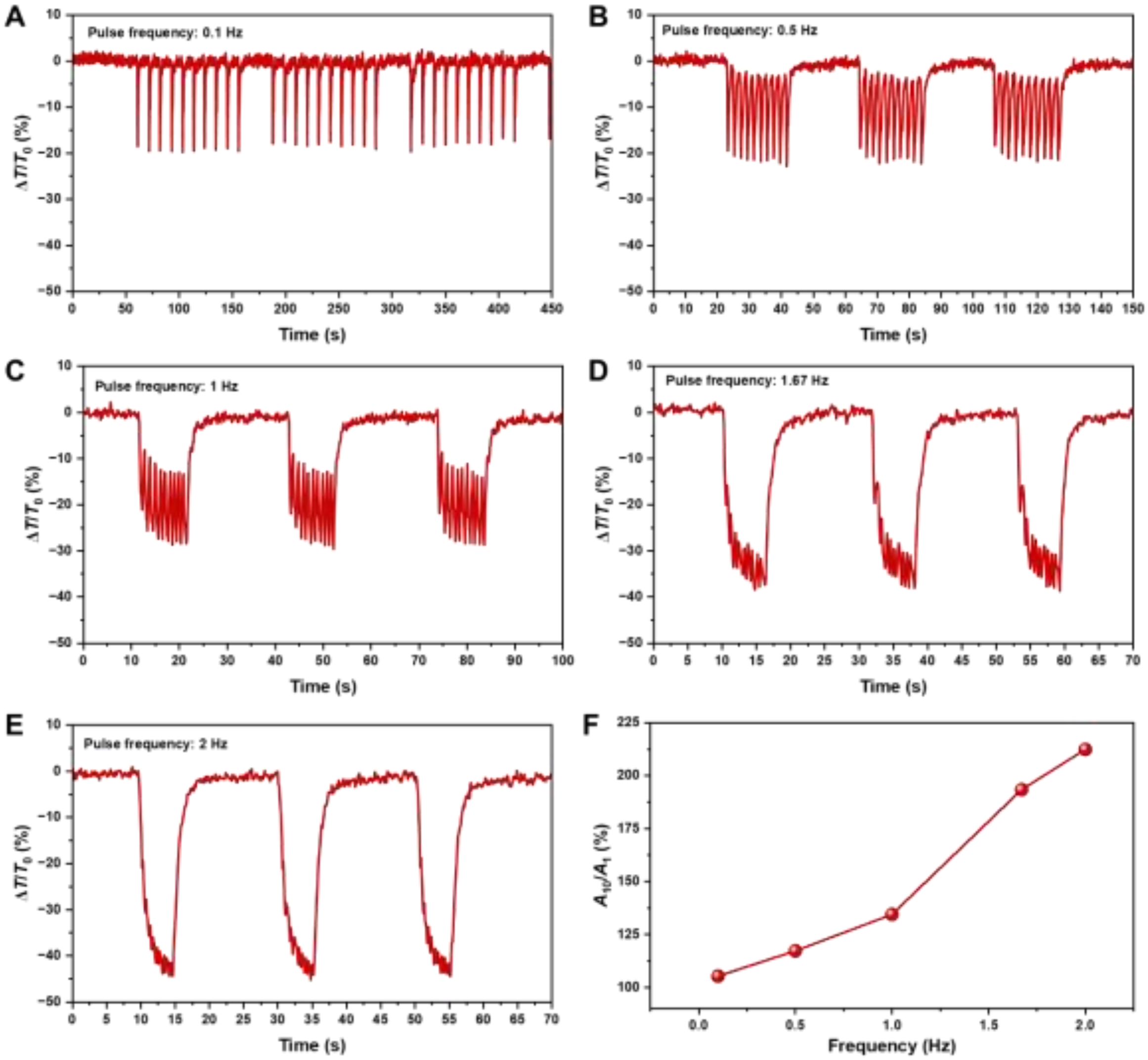

Fig. S16. Spike-rate dependent plasticity. (**A-E**) IPSPs of the EP-IDNC device triggered by 10 consecutive pulses at (A) 0.1 Hz, (B) 0.5 Hz, (C) 1 Hz, (D) 1.67 Hz, and (E) 2 Hz. Each pulse trains were repeated for 3 times. (**F**) The ratio of the 10th pulse peak response to the 1th pulse peak response as a function of pulse frequency.

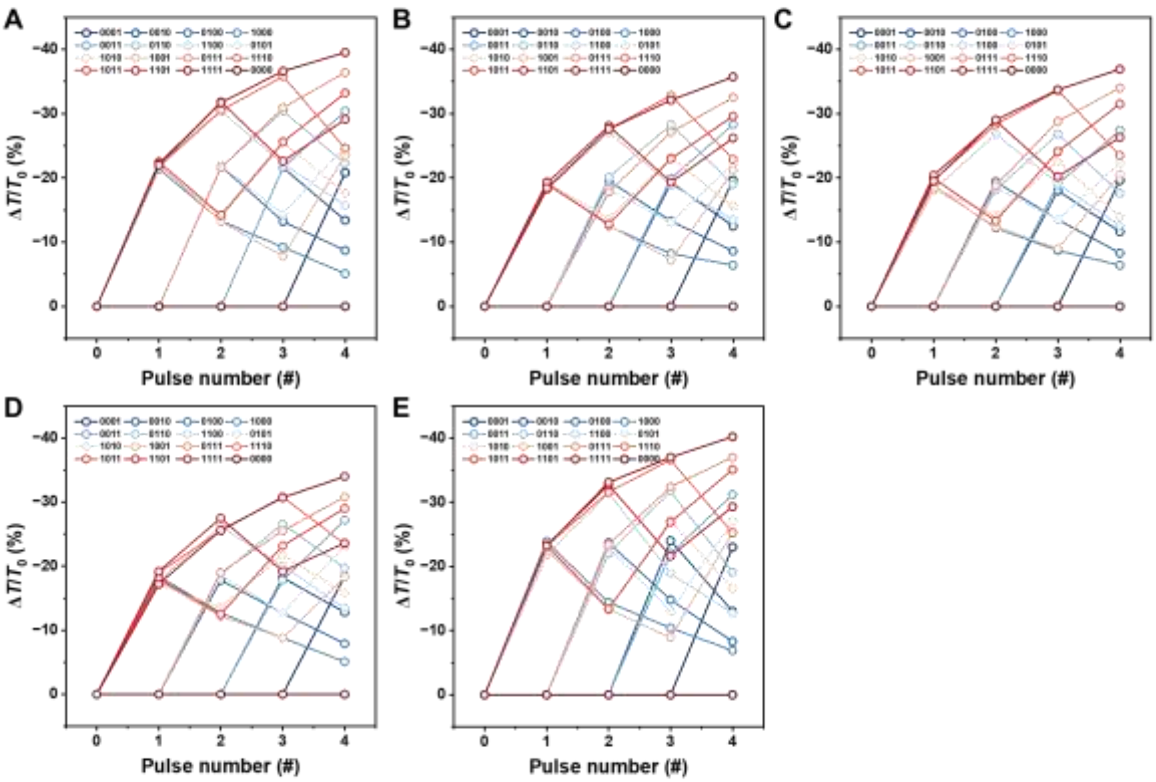

Fig. S17. Reservoir states used to differentiate different temporal inputs. (**A-E**) The transmittance response of 5 EP-IDNC devices to 16 different pulse streams, showing similar repones from all devices. When the number is “0”, a 0 V, 1 s pulse is applied to the device, while the number is “1”, a 0.7 V, 1 s pulse with 50% duty cycle is applied to device. A series resistor of 3.8 KΩ is used to control the STM behavior of the device.

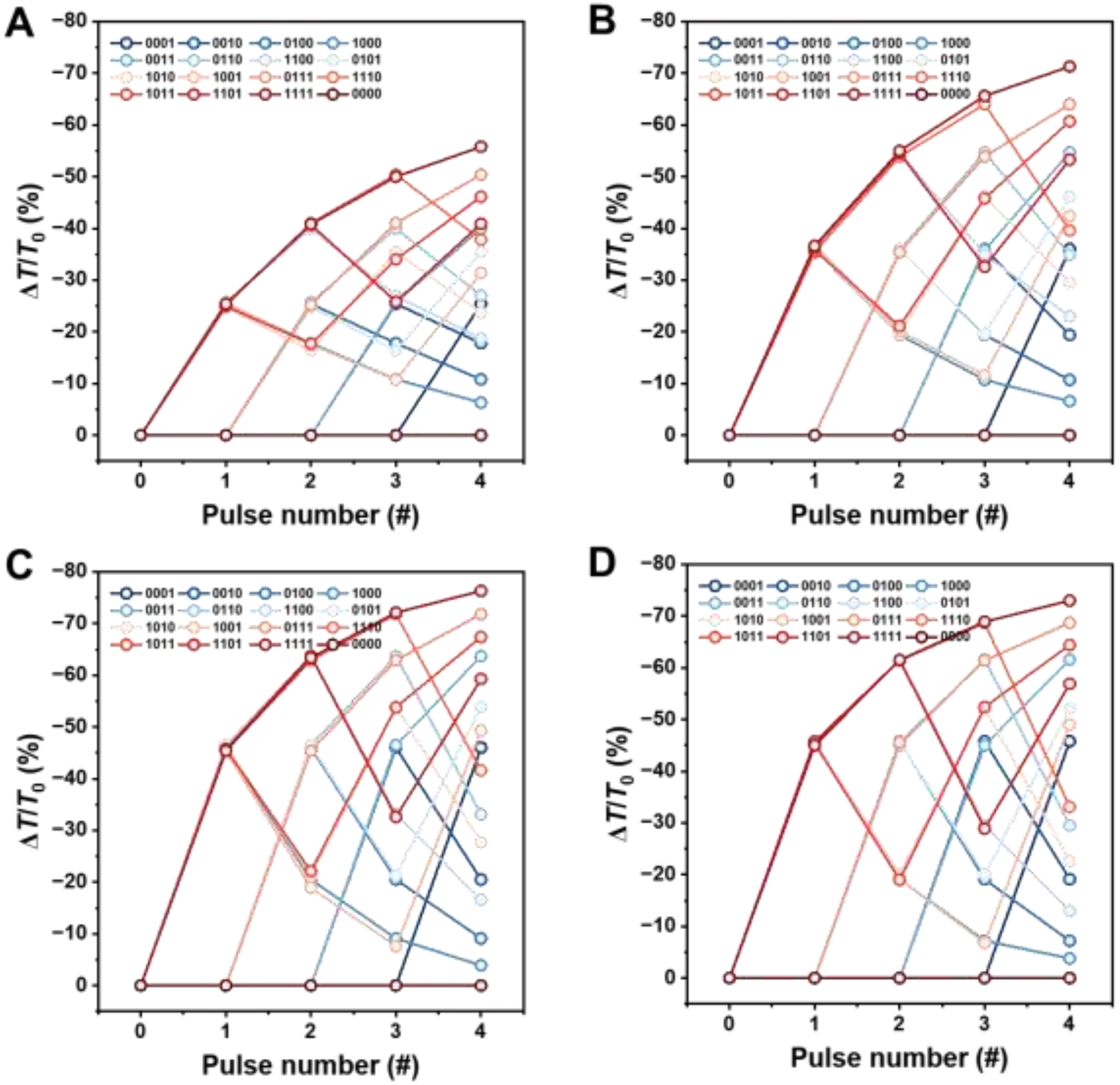

Fig. S18. Reservoir states of the EP-IDNC device with much thick electrochromic layer under different pulse stream conditions. (**A**) When the number is “0”, a 0 V, 2 s pulse is applied to the device, while the number is “1”, a 1 V, 2 s pulse with 50% duty cycle is applied to the device. (**B**) When the number is “0”, a 0 V, 2.5 s pulse is applied to the device, while the number is “1”, a 1 V, 2.5 s pulse with 60% duty cycle is applied to the device. (**C**) When the number is “0”, a 0 V, 3.5 s pulse is applied to the device, while the number is “1”, a 1 V, 3.5 s pulse with 57.14% duty cycle is applied to the device. (**D**) When the number is “0”, a 0 V, 4 s pulse is applied to the device, while the number is “1”, a 1 V, 4 s pulse with 50% duty cycle is applied to the device.

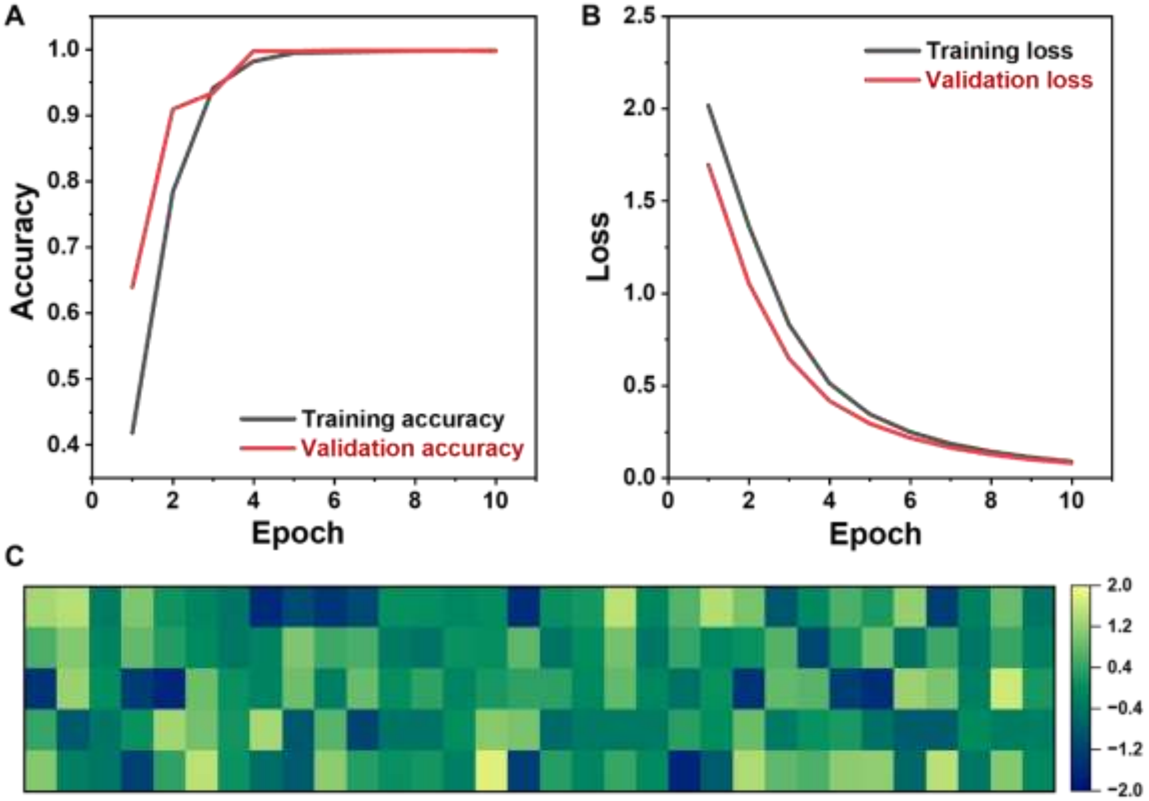

Fig. S19. Simulation results for the photonic neural network for MNIST classification. (**A**) Training and validation accuracy as a function of epoch. (**B**) Training and validation loss as a function of epoch. (**C**) Weights of readout layers after training.

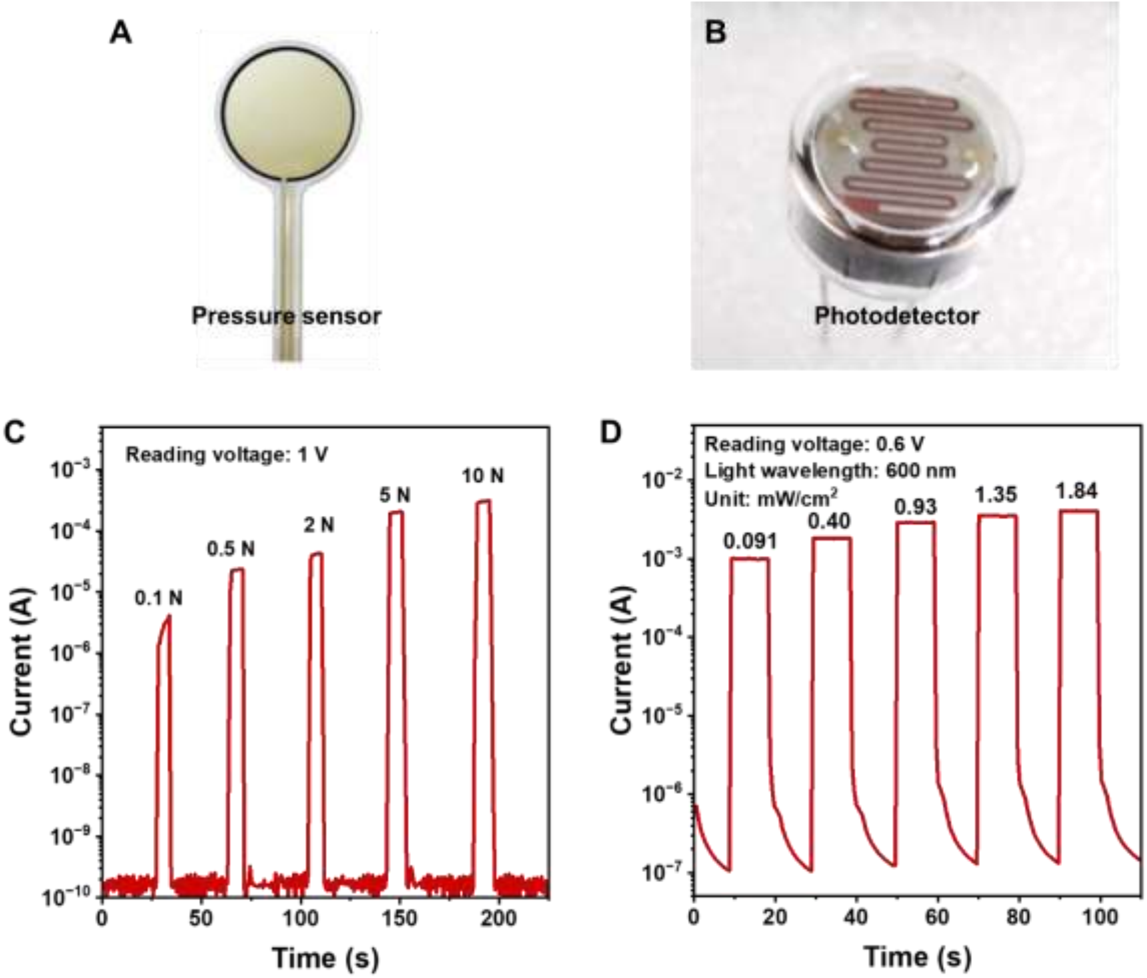

Fig. S20. Sensor performance. (**A**, **B**) Optical images of the commercially available (A) pressure sensor and (B) photodetector; (**C**, **D**) Current response of (C) the pressure sensor and (D) the photodetector under external stimuli.

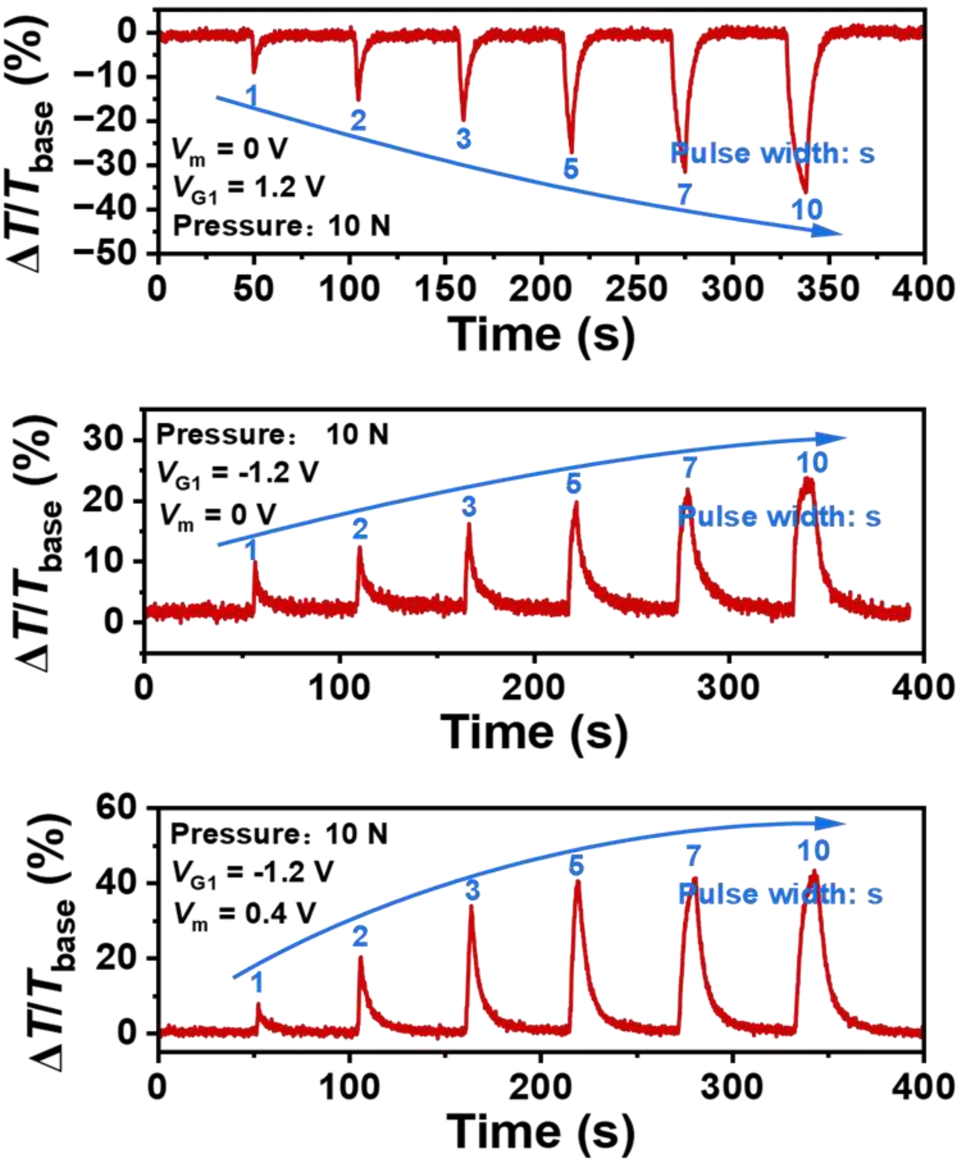

Fig. S21. The relative transmittance response of multi-terminal EP-IDNC device interfaced with the pressure sensor to six different pressure stimuli durations.

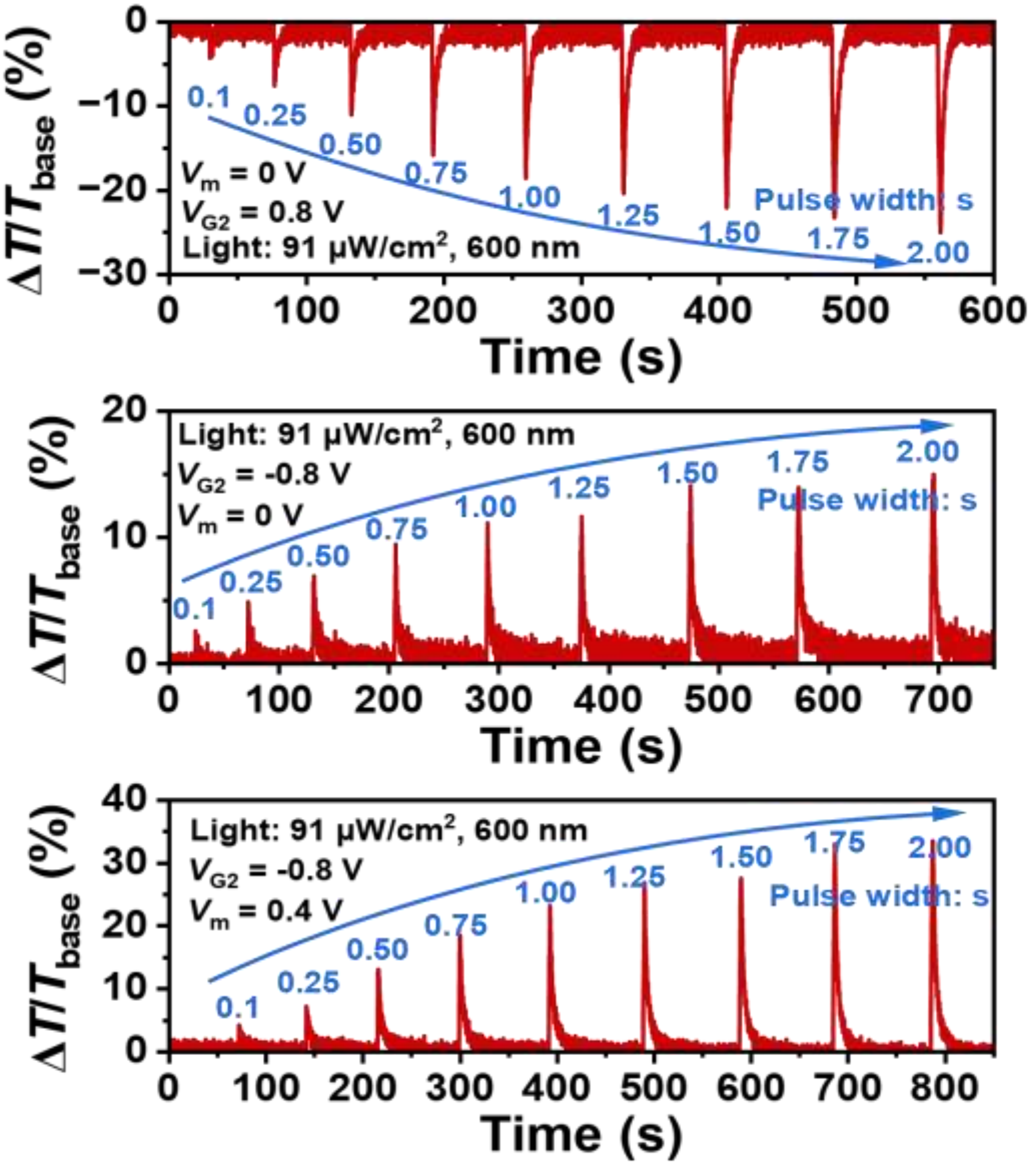

Fig. S22. The relative transmittance response of multi-terminal EP-IDNC device interfaced with the photosensor to different light stimuli duration.

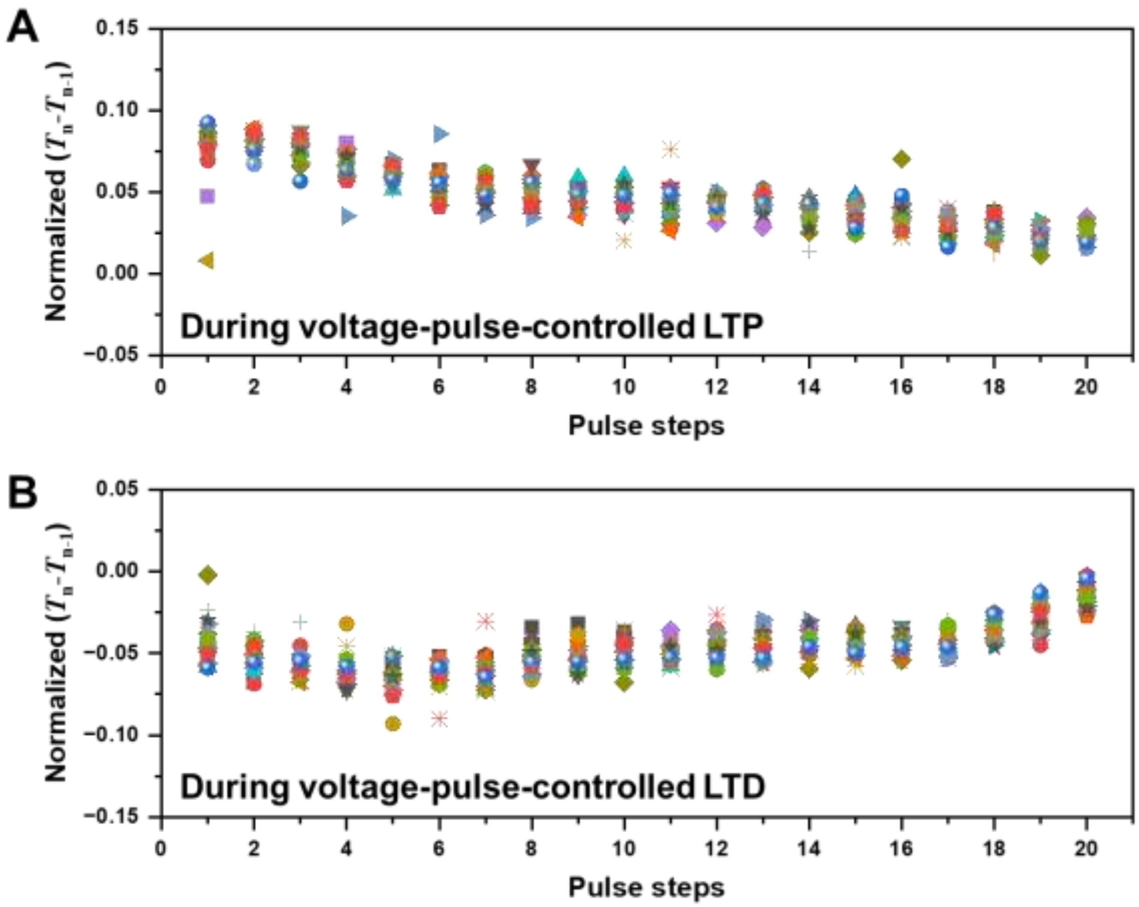

Fig. S23. Reproducibility of normalized transmittance change at each weight updating steps in the LTP-LTD cyclic curves. During the potentiation stages, 7 V pulses with a duration of 2 seconds were administered to the EP-IDNC device through a 910 KΩ resistor. Conversely, during the depression stage, -6 V pulses with a duration of 2 seconds were applied to the EP-IDNC device through a 910 KΩ resistor.

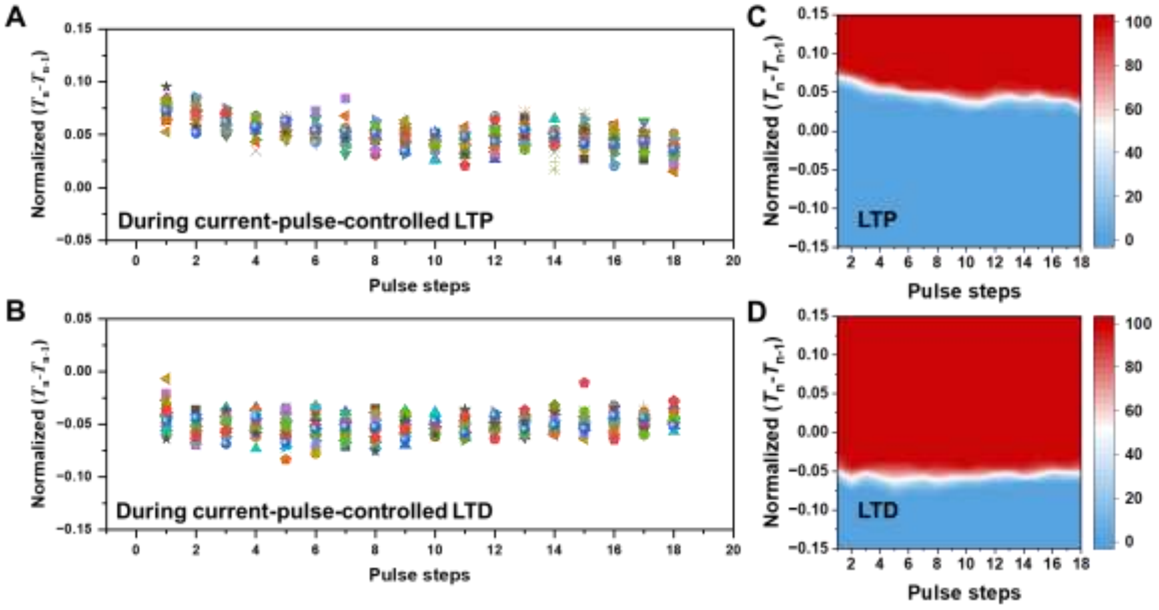

Fig. S24. Reproducibility of normalized transmittance change at each weight updating steps in the LTP-LTD cyclic curves. During the potentiation stages, -5 μA current pulses with width of 2 s were applied directly to the EP-IDNC device, while during the depression stage, 5 μA current pulses with width of 2 s were applied directly to the EP-IDNC device.

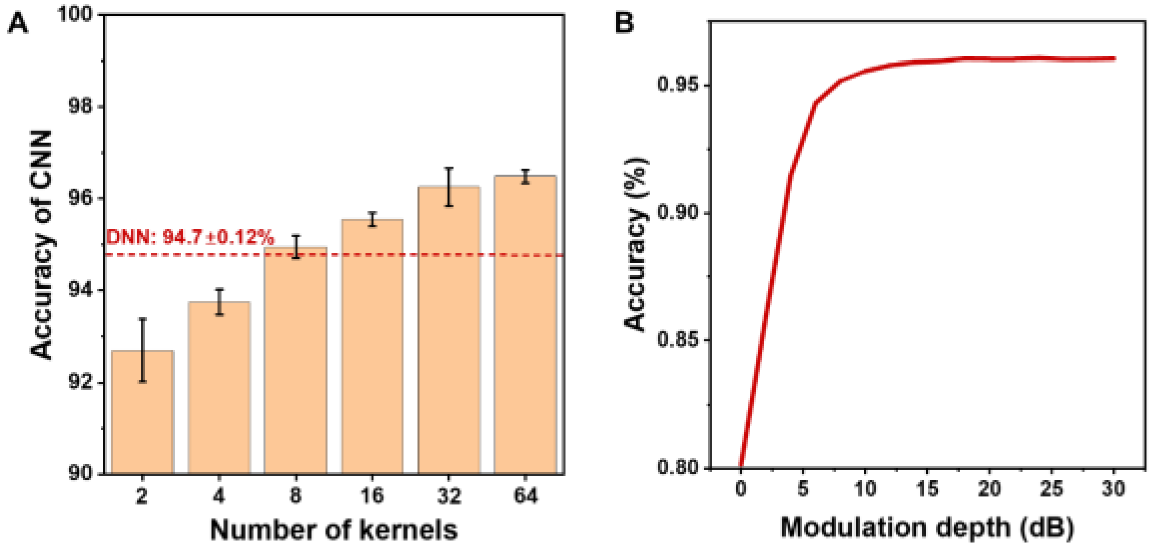

Fig. S25. The impact of convolution kernels on training accuracy. (**A**) Accuracy as a function of number of kernels. (B) Accuracy as a function of modulation depth of the EP-IDNC device-based kernels.

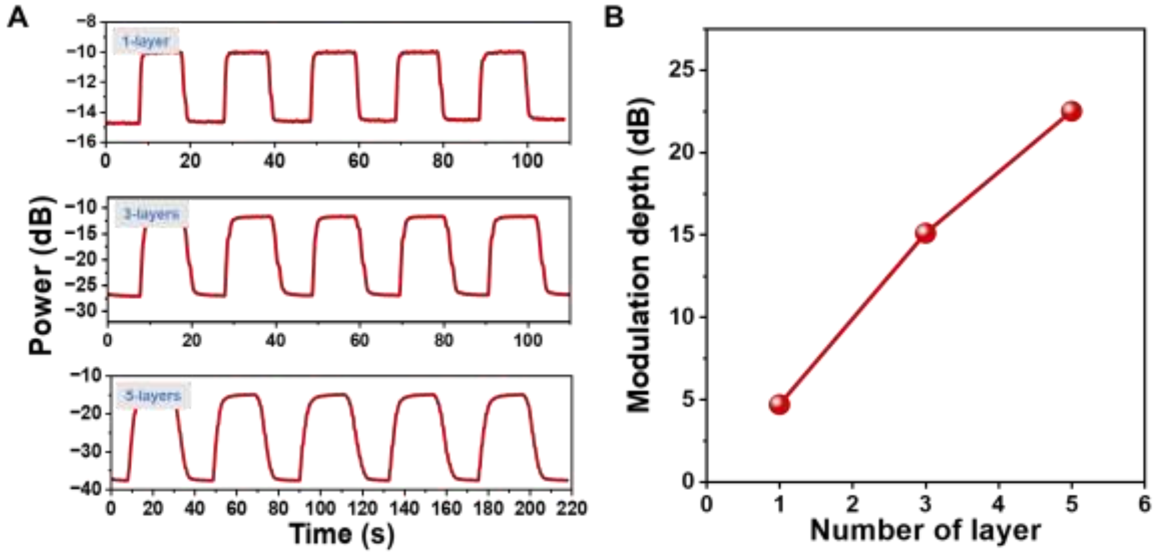

Fig. S26. Thickness controlled modulation depth. (**A**, **B**) The modulation depth can be modulated by change the thickness of the p(g2T-T) electrochromic layer in the EP-IDNC device. To adjust the thickness of the p(g2T-T) films, multiple rounds of spin-coating and UV crosslinking were utilized (refer to methods). A "1-layer" signifies that the p(g2T-T) film was obtained through a single round of spin-coating followed by UV crosslinking. Similarly, "3-layers" and "5-layers" indicate that the spin-coating and UV crosslinking process was repeated three and five times, respectively.

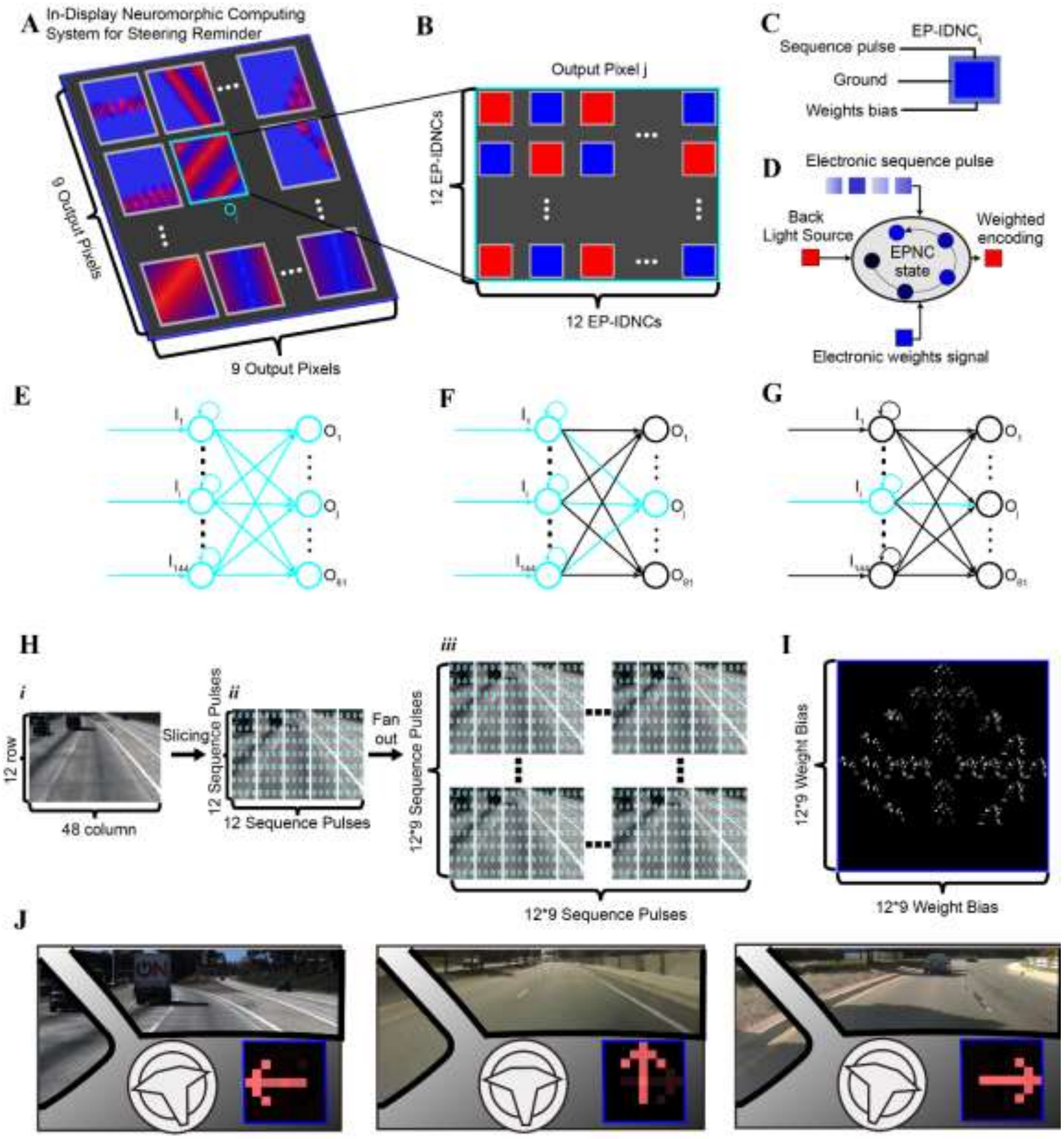

Fig. S27. Detailed illustration of the in-display neuromorphic computing system for the steering reminder task. (A) A 9×9 array of output pixels physically implements the reservoir computing architecture depicted in **(E)**. **(B)** Enlarged view of an output pixel j, containing 144 EP-IDNC devices arranged in a 12×12 array, corresponding to the cyan portion of the reservoir computing in **(F)**. **(C)** Schematic of the i-th EP-IDNC in output pixel j, labeled EP-IDNC$_{ij}$. **(D)** Each device has one terminal receiving sequence pulses and another receiving weight signal, generating weighted optical encoding as the visible output, corresponding to the cyan portion of the reservoir computing in **(G)**. **(H)** ***i.*** Road information captured by a monocular camera, divided into a 12×48 pixel array. ***ii.*** By slicing every four pixels, 12×12 input sequences are obtained, corresponding to 144 input nodes. ***iii.*** These 144 sequences are electronically fanned out to 11,664 EP-IDNC devices, along with 11,664 weight elements (**I**). The resulting biased encodings are then optically fanned in to the 9×9 output pixels to yield the final steering reminder result (**J**).

Fig. S27 shows device components and the working principles of our EP-IDNC system for simulation of steering reminder in more detail. The simulated EP-IDNC system for the steering reminder task comprises 81 output pixels arranged in a 9×9 array (**Fig. S27A**), with each output pixel contains 144 EP-IDNC devices arranged in a 12×12 array (**Figs. S27B-D**). This system physically implements the complete reservoir computing architecture (**Fig. S27E**), with each output pixel (i.e., with O_j as an example) implementing the cyan portion of the reservoir computing framework, which includes the temporal encoding and weighted operations associated with O_j (see **Fig. S27F**). **Fig. S27C** illustrates the i-th EP-IDNC in output pixel j, labeled $EP\text{-}IDNC_{ij}$. Each EP-IDNC is a multi-terminal device with one terminal receiving sequence pulses and another receiving weights signal (**Fig. 27D**). Hence, $EP\text{-}IDNC_{ij}$ physically realizes the cyan portion of the reservoir computing shown in **Fig. S27G**.

When the sensor (e.g., a monocular camera) captures the road information (**Fig. S27H(i)**), the input image is divided into a 12×48 pixel array. By slicing every four pixels into a sequence, we obtain 12×12 input sequences (**Fig. S27H(ii)**), corresponding to 144 input nodes. These 144 input sequences are electronically fanned out 81 times to drive 11,664 (144×81) EP-IDNC devices (**Fig. S27H(iii)**), in parallel with 11,664 (144×81) weight elements (**Fig. S27I**). The resulting biased encodings from these EP-IDNC devices are then optically fanned in to the 9×9 output pixels, producing the steering reminder results (**Fig.S27J**).

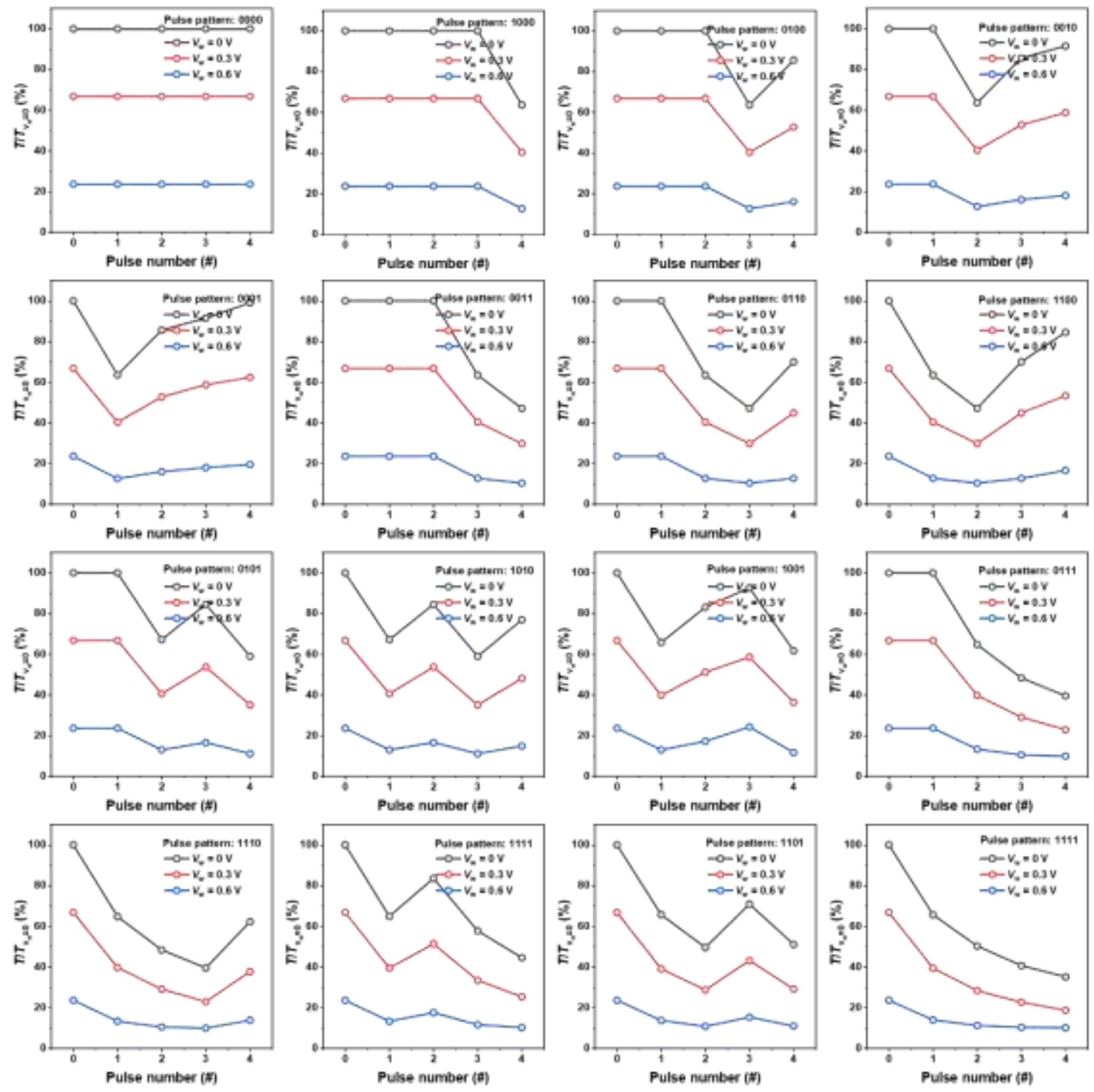

Fig. S28. The transmittance response of the triple-terminal EP-IDNC device. The transmittance is tested after exposure to different sequence pulses under varying weight control signal (V_w).

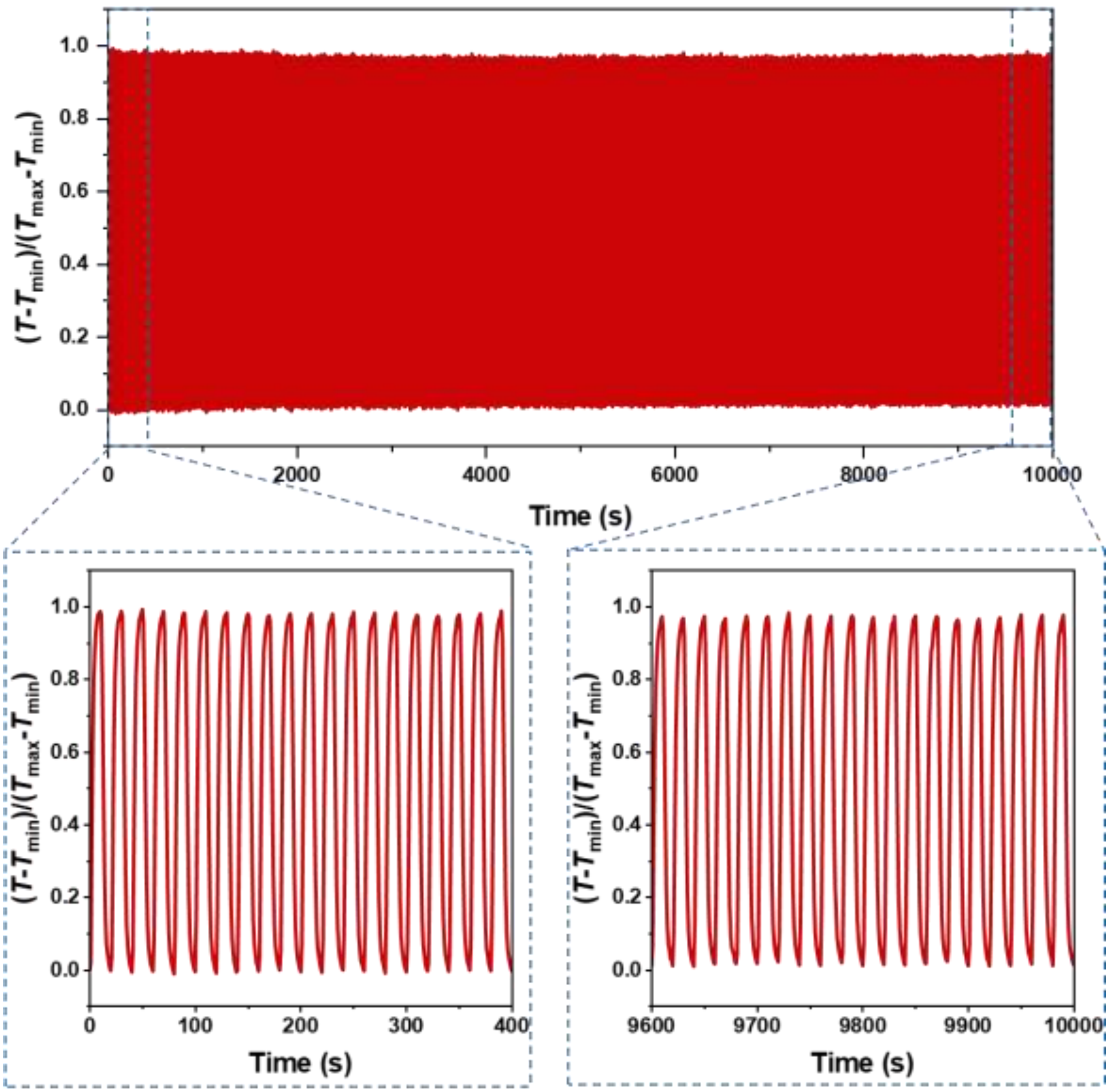

Fig. S29. Switching endurance of our EP-IDNC device, tested by applying a square wave of ±0.8 V.

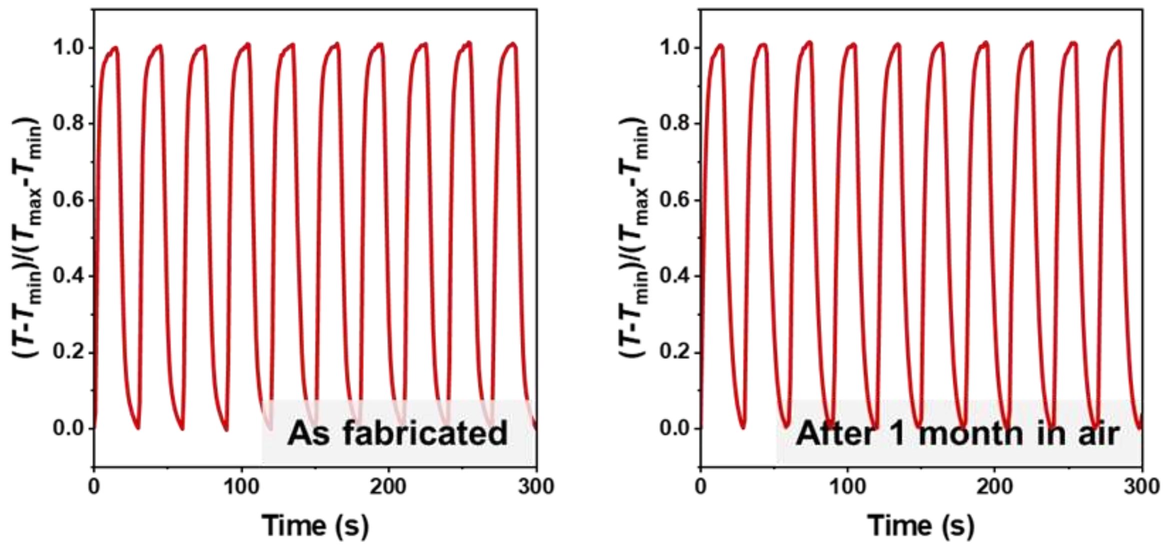

Fig. S30. Stability of our EP-IDNC device in air after PDMS encapsulation.

Table S1. Comparison of our p(g2T-T)-based EP-IDNC device with previously reported neuromorphic and typical electrochromic devices.

	Materials and structure	Organic device	Volatile memory	Non-volatile memory	Reconfigurable after fabrication	Visualization capability	Multiterminal operability	Reading wavelength	Operation voltage	In-display computing demonstration	References
**Electrical neuromorphic device**	P(g2T)-based ECRAM	√	×	√	×	×	×	N/A	<1 V	×	*Matter* 5, 3375–3390 (2022)
	P(g2T-TT)-based ECRAM	√	×	√	×	×	×	N/A	<1 V	×	*Sci. Adv.* **6**, eabb2958 (2020)
	PEDOT: PSS-based ECRAM	√	√	√	√	×	×	N/A	<1 V	×	*Nat. Mater.* **16**, 414-418 (2017)
	$HfZrO_x$ ferroelectric transistor	×	×	√	×	×	×	N/A	<6 V	×	*Sci. Adv.* **8**, eabm8537 (2022).
	$SiTe_x$-based PCM	×	×	√	×	×	×	N/A	<3 V	×	*Nature*, **628**, 293-298 (2024)
	$Ge_{15}Sb_{85}$ memtransistor	×	√	√	√	×	×	N/A	<20 V	×	*Nat. Nanotechnol.* **17**, 507-513 (2022)
	a-In_2Se_3 ferroelectric transistor	×	√	√	√	×	×	N/A	<40 V	×	*Appl. Phys. Rev.* **10**, 011408 (2023)
	Ag/PVP/Ag nanowire memristor	×	√	×	×	×	√	N/A	<8 V	×	*Nat. Mater.* **21**, 195-202 (2022)
	h-BN based Memristor	×	√ (Ag/h-BN/Pt)	√ (Au/h-BN/Au)	×	×	×	N/A	<6 V	×	*Nat. Electron.* **3**, 638-645 (2020)
**Spintronic neuromorphic devices**	Pt/GdFeCo/MgO-based synaptic device	×	×	√	×	×	×	N/A	30 mV (write) 1.8 V (access)	×	*Nat. Electron.* *3*, 148 (2020).
	Ta/W/CoFeB/MgO-based neuron	×	×	√	×	×	×	N/A	<20 V	×	NPG Asia Mater. 13, 11 (2021)
	Pt/Co/Ir-based synaptic device	×	√	×	×	×	×	N/A	Not mentioned	×	*Sci. Adv.* **8**, eabq5652 (2022)
**Photonic neuromorphic devices**	S2OC solution with anti-Stokes Photoluminescence	√	√	×	×	×	×	370 nm	N/A	×	*J. Am. Chem. Soc.* **145**, 11988−11996 (2023)
	$Ge_2Sb_2Te_5$ combined with waveguide	×	×	√	×	×	×	1570 nm	N/A	×	*Sci. Adv.* **3**, e1700160 (2017)
	$Ge_2Sb_2Te_5$ combined with waveguide	×	×	√	×	×	×	1598 nm	N/A	×	*Sci. Adv.*, **5**, eaau5759 (2019)
	Ge_2Sb_2Te combined with waveguide	×	×	√	×	×	×	1570.4 nm	<6.8 V	×	*Nat. Commun.* **14**, 2887 (2023)
	Ge_2Sb_2Se combined with planarized silicon waveguide	×	×	√	×	×	×	1550 nm	<8 V	×	*Light-Sci. Appl.* **12**, 189 (2023)
	Gallium lanthanum oxysulphide fiber	×	√	√	√	× Hasn't demonstrated	×	650 nm	N/A	×	*Adv. Optical Mater.* **3**, 635–641 (2015)
**Electrochromic devices**	Copolymer-based electrochromic device	√	×	×	×	√	×	Full visible region	<1.5 V	×	*Nat. Commun.* **15**, 8457 (2024)
	Electrochromic-based microfiber synaptic device	√	√	√	×	× Low contrast and hasn't demonstrated	×	605 nm	<3 V	×	*ACS Appl. Mater. Interfaces*, **15**, 9705-9713 (2023)
	P3HT-based electrochemical synaptic transistors	√	√	×	×	√	×	350-750 nm	<3 V	×	*Nano Lett.* **23**, 5264 (2023)
	WO_3−NiO electrochromic waveguide	×	√	√	×	×	×	1310 nm	<2 V	×	*ACS Appl. Electron. Mater.* **2**, 2057-2063 (2020)
	WO_3-based electrochromic devices	×	×	×	×	√	×	400-2500 nm	<2.4 V	×	*Nat. Electron.* **5**, 45 (2022)
	WO_3/VO_2-based electrochromic devices	×	×	×	×	√	×	400-1500 nm	<1.5 V	×	*Nat. Sustain.* 7, 796-803 (2025)
**EP-IDNC device**	**P(g2T-T)-based EP-IDNC device**	√	√	√	√	√	√	**500~750 nm**	**<1 V**	√	**This work**

Movie S1.
In-display computing and visualization for car steering reminder (simulated video).

Supplementary Reference

1. Dai Y, Dai S, Li N *et al.* Stretchable redox-active semiconducting polymers for high-performance organic electrochemical transistors. *Adv Mater* 2022; **34**: 2201178.
